# Revisiting the dihaloelimination potential of *Dehalococcoides* revealed by genomic and proteomic analyses

**DOI:** 10.1128/aem.00209-26

**Published:** 2026-05-07

**Authors:** Xiaocui Li, Hongming Cai, Hongyan Wang, Jiubin Chen, Tong Zhang, Haiwei Wei, Lian Yu, Zongming Xiu, Jun Yan, Yi Yang

**Affiliations:** 1 Key Laboratory of Forest Ecology and Silviculture, Institute of Applied Ecology, Chinese Academy of Sciences 74763 , Shenyang, Liaoning, China; 2 Key Laboratory of Pollution Ecology and Environmental Engineering, Institute of Applied Ecology, Chinese Academy of Sciences 74763 , Shenyang, Liaoning, China; 3 School of Earth System Science, Tianjin University 12605, Tianjin, China; 4 College of Environmental Science and Engineering, Nankai University 12538, Tianjin, China; 5 Department of Environmental Engineering, Beijing Institute of Petrochemical Technology 34735, Beijing, China; 6 Zhejiang Key Laboratory of Environment and Health of New Pollutants, School of Environment, Hangzhou Institute for Advanced Study, University of Chinese Academy of Sciences 74519, Hangzhou, China; 7 Shanghai Key Laboratory of Polar Life and Environment Sciences, Shanghai Jiao Tong University 12474, Shanghai, China; 8 Key Laboratory of Polar Ecosystem and Climate Change, Shanghai Jiao Tong University 12474, Shanghai, China; University of Delaware, Lewes, Delaware, USA

**Keywords:** *Dehalococcoides*, vicinal halogenated aliphatic alkanes, dihaloelimination, organohalide respiration, reductive dechlorination

## Abstract

*Dehalococcoides* (*Dhc*) has recently gained significant attention in microbial reductive dehalogenation due to its specialized ability to metabolize various halocarbons. While previous reports have documented *Dhc*-mediated dihaloelimination of 1,2-dichloroethane (1,2-DCA), 1,2-dichloropropane (1,2-DCP), and 1,2-dibromoethane, this study reveals an expansion of *Dhc*’s dihaloelimination substrate range. We successfully established PJ_DCA_ and PJ_TCA_ enrichment cultures from petroleum-contaminated soil, exhibiting dihaloelimination activity toward 1,1,2-trichloroethane (1,1,2-TCA), 1,2,3-trichloropropane (1,2,3-TCP), and 1,1,2,2-tetrachloroethane (1,1,2,2-TeCA)—substrates previously only known to be transformed by other organohalide-respiring bacteria (OHRB). Amplicon sequencing revealed the predominance of *Dhc* as the primary OHRB within the PJ_DCA_ and PJ_TCA_ cultures, leading to the identification of two novel *Dhc* populations, designated as strains PJ_DCA_ and PJ_TCA_, respectively. These strains showed robust growth yields of 4.1 ± 0.4 × 10^7^ and 7.6 ± 0.4 × 10^7^ cells per μmol Cl^−^ released when using 1,2-DCA and 1,1,2-TCA as electron acceptors, respectively. Genomic analysis revealed a reductive dehalogenase (RDase) homologous to the characterized DcpA, an enzyme known for dihaloelimination activity. Proteomic studies confirmed the expression of this DcpA-like RDase during dihaloelimination of 1,2-DCA, 1,1,2-TCA, and 1,2-DCP. This work identifies *Dhc* strains that retain dihaloelimination activity toward classical substrates while expanding this capability to 1,1,2-TCA, 1,2,3-TCP, and 1,1,2,2-TeCA. The discovery of these versatile *Dhc* strains enhances our understanding of microbial dehalogenation potential across diverse geological settings and improves prospects for bioremediation of halogenated alkane contaminants.

**IMPORTANCE:**

This study identifies *Dehalococcoides* strains capable of dihaloeliminating diverse chlorinated alkanes (1,1,2-TCA, 1,2,3-TCP, 1,1,2,2-TeCA), expanding their bioremediation potential. These compounds are persistent groundwater contaminants with high toxicity. The discovery of *Dhc* populations (PJ_DCA_/PJ_TCA_) with robust growth yields (10^7^ cells/μmol Cl^−^) and broad substrate range offers new solutions for detoxifying complex halogenated pollutant mixtures. The identification and proteomic confirmation of DcpA-like RDases (DheA) provide the genetic and functional basis for this expanded dihaloelimination capacity. These findings advance strategies for *in situ* remediation of industrial sites contaminated with C_2_-C_3_ halocarbons, reducing ecological and human health risks.

## INTRODUCTION

The improper disposal and accidental release of halogenated alkanes, such as 1,2-dichloroethane (1,2-DCA) and 1,1,2-trichloroethane (1,1,2-TCA), into the environment present significant ecological and public health concerns. These compounds exhibit notable environmental persistence, toxicity, and recalcitrance to degradation (1–3). Their tendency to form dense non-aqueous phase liquids (DNAPLs) enables long-term persistence in oxygen-depleted subsurface environments. Microbial bioremediation has been successfully implemented as an effective *in situ* treatment technology for such contaminated sites (4), with anaerobic reductive dehalogenation by organohalide-respiring bacteria (OHRB) representing the predominant degradation pathway. This energy-conserving biochemical process, wherein OHRB utilize organohalogens as terminal electron acceptors (5), has become a major focus of environmental microbiology research due to its critical role in natural attenuation and engineered remediation of halogenated contaminants.

The primary dehalogenation pathways for 1,2-DCA in anoxic environments include dichloroelimination to ethene, hydrogenolysis dehalogenation to chloroethane (CA), and dehydrohalogenation to vinyl chloride (VC). In contrast, 1,1,2-TCA can be transformed into 1,2-DCA and VC through reductive hydrogenolysis and dichloroelimination, respectively (6). To date, only five genera of OHRB have been identified as capable of reductively metabolizing 1,2-DCA and/or 1,1,2-TCA: *Dehalococcoides* (*Dhc*), *Dehalogenimonas* (*Dhgm*), *Dehalobacter* (*Dhb*), *Desulfitobacterium*, and *Trichlorobacter* (Table S1). Notably, several strains, including *Trichlorobacter* sp. strain IAE (6), *Dhb* sp. strain WL (7), *Desulfitobacterium dichloroeliminans* strain DCA1 (5, 8), and multiple *Dhgm* strains (e.g., *Dhgm lykanthroporepellens* strain BL-DC-9 (9), “*Candidatus* Dehalogenimonas loeffleri” strain W (10), *Dhgm alkenigignens* strains IP3-3, SPB-1 (11), and BRE15M (12), can conserve energy via dihaloelimination of both 1,2-DCA and 1,1,2-TCA. Among these, *Dhgm* strains and *Desulfitobacterium dichloroeliminans* strain DCA1 also exhibit the capacity to dihaloeliminate diverse vicinal chlorinated C_2_−C_3_ alkanes, including 1,1,2,2-tetrachloroethane (1,1,2,2-TeCA), 1,2-dichloropropane (1,2-DCP), 1,2,3-trichloropropane (1,2,3-TCP).

In comparison, certain bacterial strains demonstrate limited dihaloelimination activity. For instance, *Dhc* strain 11a, *Dhgm etheniformans* strain GP, *Desulfitobacterium* sp. strain AusDCA, and *Trichlorobacter* sp. strain AY have been reported to mediate 1,2-DCA dihaloelimination but show negligible activity toward 1,1,2-TCA (13–16). Additionally, *Dhc* strains 195 and BAV1 efficiently respire on 1,2-DCA, producing 99% ethene (via dichloroelimination) and 1% VC (via dehydrochlorination) (17, 18), yet neither can utilize 1,1,2-TCA as an electron acceptor. This highlights the historically narrow substrate range of *Dhc* for vicinal halogenated aliphatic alkanes and the absence of *Dhc* isolates capable of broad-spectrum dihaloelimination. This critical knowledge gap limits both our mechanistic understanding of *Dhc*’s role in natural attenuation of halogenated alkanes and the development of efficient bioremediation strategies for sites contaminated with complex halogenated alkane mixtures.

This study reports the enrichment of two novel *Dhc* strains, PJ_DCA_ and PJ_TCA_, from the soil microbiome of a petroleum-contaminated site in Northeast China. These strains are capable of dehalogenating 1,2-DCA to ethene and 1,1,2-TCA to VC, respectively. The presence of these novel *Dhc* strains was confirmed using 16S rRNA gene-targeted PCR and high-throughput amplicon sequencing. Growth kinetics analysis demonstrated a clear coupling between *Dhc* proliferation and the dehalogenation of multiple halogenated alkanes, including 1,2-DCA, 1,1,2-TCA, 1,2-dibromoethane (1,2-DBA), 1,1,2,2-TeCA, 1,2-DCP, and 1,2,3-TCP, highlighting their broad substrate range. Genomic analysis identified the reductive dehalogenases (RDases) likely responsible for dihaloelimination in strains PJ_DCA_ and PJ_TCA_, designated as DheA1 and DheA2, respectively. These RDases share 89.9%–94.8% amino acid sequence identity with characterized DcpA from *Dhgm* strain BL-DC-9 and *Dhc* strains KS and RC. Proteomic profiling of enrichment cultures dihaloeliminating 1,2-DCA, 1,1,2-TCA, and 1,2-DCP confirmed the expression of DheA in both PJ_DCA_ and PJ_TCA_ cultures. These findings expand our understanding of the metabolic diversity within the *Dhc* genus, enhancing bioremediation strategies targeting a wide range of halogenated alkane contaminants.

## MATERIALS AND METHODS

### Chemicals

1,2-DCA, 1,1,2-TCA (97% purity), 1,2-DCP, and 1,2,3-TCP were procured from Sigma-Aldrich (St. Louis, MO, USA). Ethene and VC were sourced from Dalian Special Gases (Dalian, Liaoning, China). Additionally, 1,1,1-trichloroethane (1,1,1-TCA) and chloroform (CF) were obtained from XIYA REAGENT (Linyi, Shandong, China) and CASMART (Beijing, China), respectively. Tetrachloroethylene (PCE) and trichloroethylene (TCE) were purchased from Macklin (Shanghai, China), while 1,2-DBA and *cis*-1,2-dichloroethene (*c*DCE) were acquired from TCI (Shanghai, China). Furthermore, 1,1,2,2-TeCA and *trans*-1,2-dichloroethene (*t*DCE) were supplied by Innochem (Beijing, China) and Mreda (Beijing, China), respectively. All chlorinated solvents, except for 1,1,2-TCA, exhibited a purity level of ≥98%. All other chemicals employed in this study were of analytical grade or higher.

### Microcosm setup and enrichment cultures

Soil samples were collected from a petroleum-contaminated site located in Panjin City, Liaoning Province, Northeast China (41.1948°N, 122.2054°E). Microcosms were established within an anaerobic glovebox (Coy Laboratory Products, Ann Arbor, MI, USA) by dispensing approximately 5 g of the petroleum-contaminated soil into 120-mL serum bottles. Each bottle contained a N_2_/CO_2_ headspace (80/20, vol/vol) and 80 mL of sterilized reduced anaerobic mineral medium (30 mM bicarbonate-buffered, pH 7.2) (19), supplemented with 5 mM lactate and the Wolin vitamin mix (19) (containing 50 μg L^−1^ vitamin B_12_). The bottles were sealed with new blue butyl rubber stoppers and crimped with aluminum caps. Subsequently, 10 mL of H_2_ was injected into the headspace of each serum bottle using gas-tight syringes. Neat 1,2-DCA (*ca*. 6 μL or 76.0 μmol, 0.9 mM aqueous concentration) or 1,1,2-TCA (*ca*. 6 μL or 64.5 μmol, 0.8 mM aqueous concentration) was added as an electron acceptor. Following the complete dehalogenation of 1,2-DCA to ethene or 1,1,2-TCA to VC, repeated transfers (3.75%, vol/vol) to the same growth medium were performed to develop solid-free enrichment cultures, designated as PJ_DCA_ (1,2-DCA-dehalogenating) and PJ_TCA_ (1,1,2-TCA-dehalogenating), respectively. Autoclaved cultures amended with 1,2-DCA or 1,1,2-TCA served as sterile controls. All serum bottles were incubated at 30°C under dark, static conditions. The reductive dehalogenation capabilities of the PJ_DCA_ and PJ_TCA_ enrichment cultures were evaluated against a range of chlorinated alkanes (1,2,3-TCP, 1,2-DCP, 1,1,2,2-TeCA, 1,1,1-TCA, and CF), chlorinated alkenes (PCE, TCE, *c*DCE, *t*DCE, and VC), and 1,2-DBA. Experiments were conducted in 120-mL serum bottles, each containing 80 mL of basal medium.

### Metagenome sequencing, assembly, and binning

Genomic DNA was extracted from soil samples, 11th-generation PJ_DCA_ enrichment culture, and 9th-generation PJ_TCA_ enrichment culture using the CTAB protocol as recommended by the Joint Genome Institute (JGI, Walnut Creek, CA, USA) (20). Sequencing libraries were prepared using the NovaSeq 6000 S4 kit (Illumina, Inc., San Diego, CA, USA), and metagenomic sequencing was performed on a NovaSeq 6000 sequencer. Raw sequence data were processed using Readfq (V8, https://github.com/cjfields/readfq) to obtain high-quality filtered reads for downstream analysis. After quality trimming and filtering, a total of 15,864,450, 30,009,186, and 30,195,742 paired-end reads were obtained from the soil, PJ_DCA_, and PJ_TCA_ samples, respectively. These reads were assembled using the JGI Metagenome Assembly Pipeline (https://github.com/kbaseapps/jgi_mg_assembly). Taxonomic classification of the metagenomic short reads was performed using Kaiju (v 1.7.3) (21). Metagenome-assembled genomes (MAGs) are generated by binning contigs into taxonomic groups using Maxbin (v2.2.4) (22). The quality of the MAGs was assessed using CheckM, which evaluates completeness and contamination based on lineage-specific marker genes (23). Taxonomic affiliation of the MAGs was determined using the GTDB-Tk software toolkit (24) and the Type (Strain) Genome Server (TYGS, https://tygs.dsmz.de) (25). According to the Metagenome Assembled Genome (MIMAG) standards proposed by the Genomic Standards Consortium, MAGs with >50% completeness and <10% contamination were classified as medium-quality, while those with >90% completeness, <5% contamination, and the presence of all rRNA genes (23S, 16S, 5S) and at least 18 tRNAs were designated as high-quality (26). High-quality MAGs derived from the PJ_DCA_ and PJ_TCA_ enrichment cultures contained draft genome sequences of *Dhc* strains, designated as PJ_DCA_ and PJ_TCA_, respectively. These draft genomes were annotated using PATRIC (v3.6.12) (http://patricbrc.org) with default parameters (27).

### Proteomics analysis

Following complete dehalogenation of 0.9 mM 1,2-DCA (PJ_DCA_ culture), 0.8 mM 1,1,2-TCA (PJ_DCA_ culture), and 0.7 mM 1,2-DCP (PJ_TCA_ culture) (aqueous concentrations), biomass from individual 480 mL enrichment cultures was aseptically harvested via centrifugation (9,000 × *g*, 10 min, 4°C). The PJ_TCA_ culture grown on 1,2-DCP was selected for proteomics as it exhibited the highest dehalogenation rate (21.1 μM day^−1^) among the vicinal halogenated aliphatic alkanes beyond the primary substrates (1,2-DCA, 45.2 μM day^−1^; 1,1,2-TCA, 29.9 μM day^−1^), providing a representative and active system to elucidate the broader substrate metabolism. The collected cells were submitted to Shanghai Bioprofile Technology Company Ltd., China, for protein identification. Trypsin-digested peptides were desalted on C18 Cartridges (Empore SPE Cartridges, Sigma-Aldrich, USA) and concentrated by vacuum centrifugation. The peptides were reconstituted in 10 μL of 0.1% (vol/vol) formic acid (HPLC Grade, Sigma-Aldrich, USA) prior to analysis. Mass spectrometry was performed using data-dependent acquisition (DDA) on a Q Exactive HF mass spectrometer coupled with an Easy nLC system (Thermo Scientific, USA). Samples were injected into a Trap Column (100 µm × 20 mm, 5 µm, C18, Dr. Maisch GmbH) and separated on a chromatographic analysis column (75 µm × 150 mm, 3 µm, C18, Dr. Maisch GmbH) at a flow rate of 300 nL/min. Mobile phase A consisted of 0.1% formic acid in water, while mobile phase B comprised 0.1% formic acid in 80% acetonitrile. The gradient profile was as follows: 0–2 min, 2%–5% B; 2–44 min, 5%–28% B; 44–51 min, 28%–40% B; 51–53 min, 40%–100% B; 53–60 min, 100% B. MS data for the PJ_DCA_ (1,2-DCA-dehalogenating) and PJ_TCA_ (1,1,2-TCA- or 1,2-DCP-dehalogenating) cultures were searched against strain-specific protein databases for *Dhc* strains PJ_DCA_ (Accession: ASM3792857v1) and PJ_TCA_ (Accession: ASM3792860v1), respectively, using MaxQuant software version 2.0.1.0. The search parameters included trypsin enzyme specificity; precursor mass tolerances of 4.5 ppm (main search) and 20 ppm (first search); variable modifications of methionine oxidation and N-terminal acetylation; carbamidomethylation as a fixed modification; target-reverse database pattern; and allowance for up to two missed cleavages. Peptide-spectrum matches (PSMs) were filtered at a false discovery rate (FDR) ≤ 0.01, and identified proteins were accepted at an FDR ≤ 0.01.

### Analytical methods

Aqueous samples (1 mL) collected from cultivation vessels were analyzed for chlorinated compounds, propene, and ethene using an Agilent GC7697A headspace autosampler coupled to an Agilent GC7890B gas chromatograph (Agilent Technologies, Santa Clara, CA, USA). The system was equipped with a flame-ionization detector (FID) and an Agilent DB-624 column (60 m × 0.32 mm × 1.8 µm), following established protocols (6). The GC inlet and FID temperatures were maintained at 200°C and 300°C, respectively. The oven temperature program was initiated at 60°C for 2 min, followed by a ramp to 200°C at a rate of 25°C/min, and a final hold at 200°C for 1 min. Standard curves were generated by injecting known quantities of each compound into serum bottles with a 40/80 (vol/vol) headspace-to-liquid ratio, replicating the conditions of the microcosm and incubation vessels. Concentrations in the aqueous or gas phase were determined by normalizing peak areas to the standard curves, and Henry’s law was applied for mass balance calculations (28). Total chlorinated compound amounts were calculated by summing the respective liquid and headspace concentrations, each multiplied by their respective volumes. Dehalogenation rates were estimated based on the concentration changes of chlorinated electron acceptors during the dehalogenation process. Growth yields were calculated as the increase in cell numbers divided by total chloride ions released, using the consumption estimates of 1,2-DCA and 1,1,2-TCA during dehalogenation (29).

## RESULTS

### The enrichment cultures PJ_DCA_ and PJ_TCA_ dihaloeliminates vicinal halogenated aliphatic alkanes

**Fig 1:**
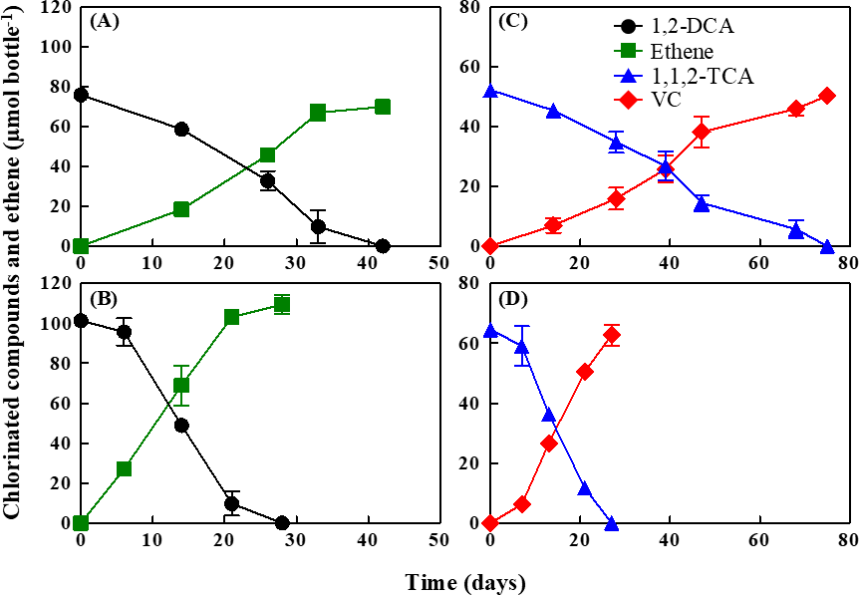
Reductive dehalogenation of 1,2-DCA and 1,1,2-TCA in the PJ_DCA_ and the PJ_TCA_ enrichment cultures, respectively. (**A and B**) illustrate the dihaloelimination of 1,2-DCA to ethene in the 6th- and 11th-generation PJ_DCA_ enrichment cultures, respectively. (**C and D**) depict the dihaloelimination of 1,1,2-TCA to VC in the 2nd- and 11th-generation PJ_TCA_ enrichment cultures, respectively. The error bars represent the standard deviations of triplicate samples and are not shown when smaller than the symbol.

In soil microcosms derived from a petroleum-contaminated site, approximately 76.0 μmol (*ca*. 0.9 mM aqueous concentration) of 1,2-DCA was dehalogenated to ethene within a 2-month period. Successive transfers of the PJ_DCA_ enrichment cultures maintained this dehalogenation capability, with rates increasing from 22.6 μM day^−1^ in the 6th-generation culture (Fig. 1A) to 45.2 μM day^−1^ in the 11th-generation culture (Fig. 1B). The absence of VC in the PJ_DCA_ enrichment cultures indicated that the microbial community employs a direct dihaloelimination mechanism to convert the 1,2-DCA directly to ethene, bypassing the formation of VC. In contrast, in the soil microcosms supplemented with 1,1,2-TCA, the only dehalogenation product was VC. The second-generation PJ_TCA_ enrichment culture transformed 53.8 μmol (*ca*. 0.7 mM aqueous concentration) of 1,1,2-TCA to VC within 75 days (Fig. 1C). By the 11th generation, PJ_TCA_ enrichment culture exhibited enhanced activity, completely dehalogenating approximately 64.5 μmol (*ca*. 0.8 mM aqueous concentration) of 1,1,2-TCA in 27 days, achieving a dehalogenation rate of 29.9 μM day^−1^ (Fig. 1D). Sterile control showed no detectable dihaloelimination products (i.e., VC, ethene) or significant changes in 1,2-DCA or 1,1,2-TCA concentrations (Fig. S1).

**Fig 2:**
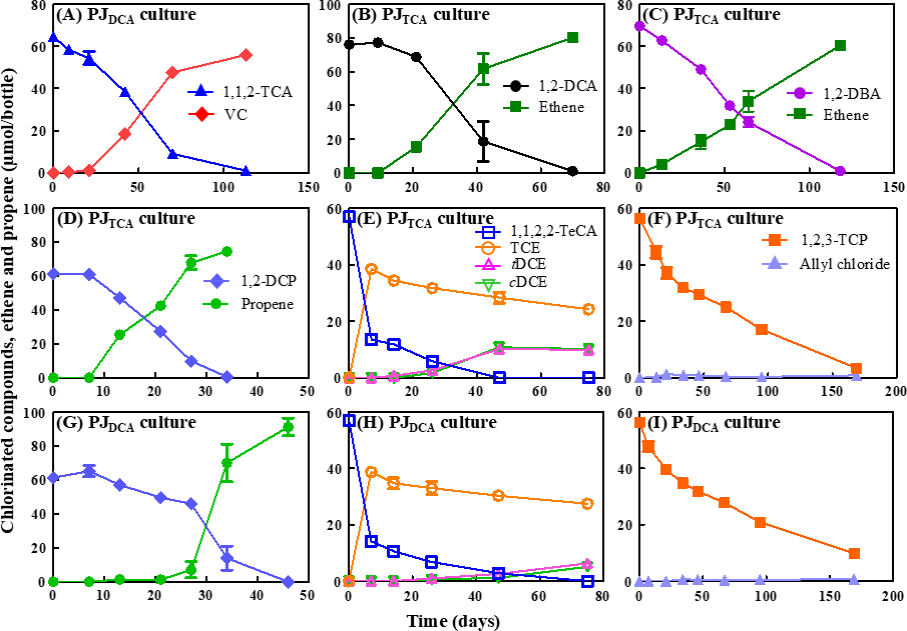
Reductive dehalogenation of 1,1,2-TCA (**A**), 1,2-DCA (**B**), 1,2-DBA (**C**), 1,2-DCP (**D and G**), 1,1,2,2-TeCA (**E and H**), and 1,2,3-TCP (**F and I**) by PJ_DCA_ cultures (AGHI) and PJ_TCA_ cultures (BCDEF). Error bars represent standard deviation of triplicate bottles.

Cross-substrate experiments revealed that PJ_DCA_ and PJ_TCA_ enrichment cultures could dihaloeliminate 1,1,2-TCA and 1,2-DCA, respectively (Fig. 2A and B). Both cultures also transformed other vicinal halogenated aliphatic alkanes: 1,2-DBA to ethene (Fig. 2C), 1,2-DCP to propene (Fig. 2D and G), 1,1,2,2-TeCA to *c*DCE/*t*DCE (Fig. 2E and H), 1,2,3-TCP to allyl chloride (Fig. 2F and I). Notably, abiotic transformation of 1,1,2,2-TeCA to TCE was observed in sterile controls, consistent with previously reported non-biological pathways (6), confirming that *c*DCE and *t*DCE originated solely from 1,1,2,2-TeCA dihaloelimination. Allyl chloride, the dihaloelimination product of 1,2,3-TCP, was detected in minimal quantities despite significant 1,2,3-TCP depletion (Fig. 2F and I). This discrepancy is explained by the rapid abiotic transformation of allyl chloride in the medium, as confirmed in a dedicated control experiment (Fig. S2). This abiotic process yielded allyl alcohol, diallyl sulfide, and diallyl disulfide as primary products, consistent with the metabolite profile observed during biological dehalogenation of 1,2,3-TCP (Fig. 2F and I) and with prior findings (6). Neither PJ_DCA_ nor PJ_TCA_ enrichment culture demonstrated dehalogenation activity with CF, 1,1,1-TCA, *c*DCE, *t*DCE, VC, PCE, or TCE, revealing substrate specificity for vicinal halogenated aliphatic alkanes.

### Community structures of soil microcosms and enrichment cultures

Taxonomic analysis at the phylum level (Table S2) using 16S rRNA amplicon sequencing revealed that *Bacteroidota*, *Bacillota*, *Pseudomonadota*, *Chloroflexota*, and *Synergistota* dominated the soil microcosms, collectively representing 88.2%–88.6% of the total sequences in the soil microcosms that completely dehalogenated 1,2-DCA or 1,1,2-TCA. In the enrichment cultures, *Bacillota* (31.6%–44.9%) and *Bacteroidota* (27.4%–58.0%) remained predominant, while *Pseudomonadota* abundances decreased significantly from 8.2% and 7.7% in the microcosms to 0.01% and 0.2% in the PJ_DCA_ and PJ_TCA_ enrichment cultures, respectively. Similarly, *Chloroflexota* abundances declined from 4.7% and 9.8% in microcosms to 0.3% and 0.6% in the PJ_DCA_ and PJ_TCA_ enrichment cultures. *Synergistota* abundances remained stable across both microcosms and enrichment cultures. Other phyla with relative abundances >1.5% included *Actinobacteriota* (2.5%–2.9%) and *Cloacimonadota* (1.5%–2.3%) in the microcosms and *Cloacimonetes* (2.1%–4.4%) in the enrichment cultures.

**Fig 3:**
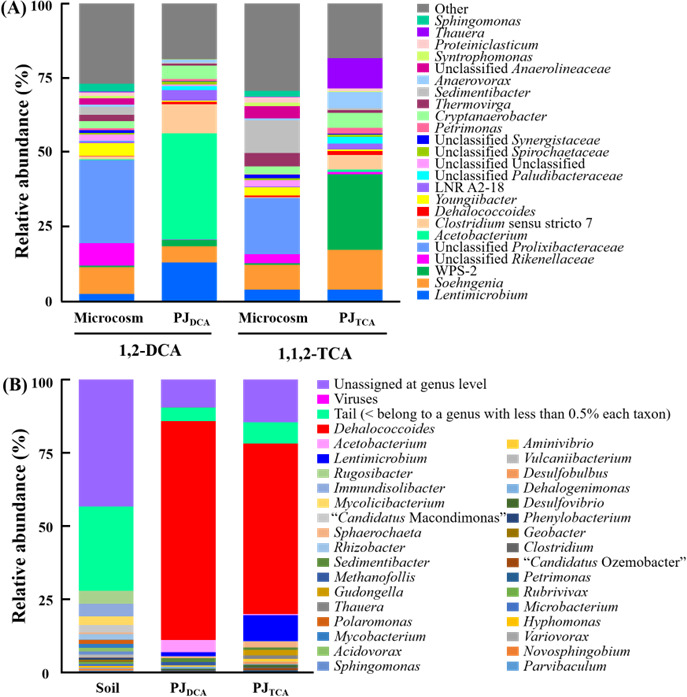
Microbial community structures at the genus level. (**A**) illustrates the microbial community structures based on 16S rRNA gene amplicon sequencing data in the microcosms and the PJ_DCA_ (14th generation) and PJ_TCA_ (11th generation) enrichment cultures amended with lactate, as well as the PJ_TCA_ (3rd generation) enrichment cultures amended with acetate, subsequent to the complete attenuation of 1,2-DCA or 1,1,2-TCA, respectively. (**B**) presents the microbial community structures based on metagenomic data from the petroleum-contaminated soil and the PJ_DCA_ (11th generation) and PJ_TCA_ (9th generation) enrichment cultures following the total consumption of 1,2-DCA and 1,1,2-TCA, respectively. The term “Others” encompasses OTUs affiliated with low-abundance genera and OTUs showing no significant difference in abundance between the microcosms and the enrichment cultures. The red frame box highlights the increase in *Dhc* abundances within the enrichment cultures.

At the genus level, unclassified *Prolixibacteraceae* (*Bacteroidota*) dominated the soil microcosms, accounting for 18.9%–28.0% of sequences (Fig. 3A). However, these taxa were either absent or present at <0.1% in the enrichment cultures. In contrast, *Acetobacterium* and WPS-2 emerged as the most abundant genera in the PJ_DCA_ and PJ_TCA_ enrichment cultures, respectively. Combined abundances of *Soehngenia* and *Lentimicrobium* rose from 11.3%–12.1% in the microcosms to 17.0%–18.2% in the enrichment cultures. *Clostridium sensu* stricto 7 increased from 0.3% to 1.0% in the microcosms to 4.9%–9.9% in the enrichment cultures. *Dhc* OTUs accounted for 0.2%–0.4% of sequences in the microcosms, increasing to 0.7% and 1.4% in the PJ_DCA_ and PJ_TCA_ enrichment cultures, respectively. To further optimize *Dhc* enrichment, we replaced lactate with acetate as carbon source. After three consecutive transfers, *Dhc* abundance in the acetate-amended enrichment cultures reached 5.9% (Fig. 3A), though the dehalogenation rate was relatively slow—complete dehalogenation of 64.54 μmol 1,1,2-TCA required 128 days (Fig. S3). Notably, no other OHRB were detected, suggesting that *Dhc* is responsible for the dihaloelimination 1,2-DCA and 1,1,2-TCA. Current research indicates that *Dhc* strains typically maintain only a single copy of the 16S rRNA gene in their genomes, whereas many other bacteria commonly found in enrichment cultures possess multiple copies. This variation in 16S rRNA gene copy number (GCN) among different taxa is a well-known factor that can bias the interpretation of 16S rRNA gene amplicon sequencing data, potentially leading to an underestimation of the relative abundance of low-GCN organisms like *Dhc* when compared to methods based on whole-genome coverage (e.g., metagenomics) (30, 31).

Metagenomic analysis further demonstrated a dramatic increase in *Dhc* abundance, from <0.001% in the original petroleum-contaminated soil to 75.4% and 59.0% in the PJ_DCA_ and PJ_TCA_ enrichment cultures, respectively (Fig. 3B). Among non-OHRB populations, *Lentimicrobium* (1.3%–9.0%) and *Acetobacterium* (0.3%–4.3%) were the dominant contributors to the PJ_DCA_ and PJ_TCA_ enrichment cultures. Notably, the substantial discrepancy between the amplicon (~1%) and metagenomic (60%–75%) estimates of *Dhc* relative abundance primarily stems from their different normalization principles (GCN vs genome coverage) and the community simplification during enrichment. Collectively, the substantial enrichment of *Dhc* populations, as evidenced by both amplicon sequencing and metagenomic data, underscores their critical role in the dihaloelimination of 1,2-DCA and 1,1,2-TCA. The absence or low abundance of other characterized dechlorinators further highlights the unique functional niche occupied by *Dhc* in these enrichment cultures.

### Growth of *Dhc* strains coupled to dihaloelimination of vicinal halogenated aliphatic alkanes

Upon complete dehalogenation of 101.3 μmol 1,2-DCA, strain PJ_DCA_ cell densities increased 125.0-fold, from an initial concentration of 8.5 ± 1.5 × 10^5^ cells mL^−1^ to 1.1 ± 0.1 × 10^8^ cells mL^−1^ (Table 1). Similarly, during the dihaloelimination of 1,1,2-TCA to VC at a rate of 18.76 μM day^−1^, strain PJ_TCA_ cell numbers rose 121.6-fold, from 1.0 ± 0.1 × 10^6^ to 1.2 ± 0.1 × 10^8^ cells mL^−1^ (Table 1). In contrast, negligible growth was observed for both strains PJ_DCA_ and PJ_TCA_ in control cultures lacking 1,2-DCA or 1,1,2-TCA (Table 1). The growth yields of strains PJ_DCA_ and PJ_TCA_ were calculated as 4.1 ± 0.4 × 10^7^ and 7.6 ± 0.4 × 10^7^ cells per μmol Cl^−1^ released, respectively (Table 1). Notably, the growth yield of strain PJ_DCA_ exceeded those of other OHRB capable of 1,2-DCA dehalogenation, such as *Dhgm*, *Dhb*, and *Trichlorobacter*, by approximately an order of magnitude (6). The strict dependence of *Dhc* growth on the presence of specific organohalide electron acceptors and the quantitative correlation between cell increase and chloride release provide direct evidence that these *Dhc* strains conserve energy via the dihaloelimination respiration of 1,2-DCA and 1,1,2-TCA. In addition, both strains demonstrated the ability to utilize a broad range of vicinal halogenated aliphatic alkanes as electron acceptors. Following complete dehalogenation of 1,2-DCP, 1,1,2,2-TeCA, 1,2,3-TCP, and 1,2-DBA, cell densities of strains PJ_DCA_ and PJ_TCA_ increased by 42- to 145-fold compared to initial inocula (Table 1). This metabolic versatility expands the known substrate range of *Dhc* and underscores its potential for bioremediation of sites contaminated with mixed halogenated aliphatic compounds.

**TABLE 1:** Growth of *Dhc* in PJ_DCA_ and PJ_TCA_ enrichment cultures amended with different electron acceptors

OHRB	Substrate consumption (μmol bottle^−1^)^*a*^	Enrichment culture: dehalogenation progress	Cell density (cells mL^−1^)^*b*^		Fold increase^*d*^	Growth yield^*e*^
			Initial	Final^*c*^		
Strain PJ_DCA_	101.30 ± 0.00	PJ_DCA_: 1,2-DCA→ETH (11th)	8.48 ± 1.48 × 10^5^	1.06 ± 0.09 × 10^8^	125.0	4.14 ± 0.37 × 10^7^
	63.46 ± 0.26	PJ_DCA_: 1,1,2-TCA→VC	1.20 ± 0.31 × 10^5^	2.18 ± 0.03 × 10^7^	181.7	1.37 ± 0.02 × 10^7^
	46.57 ± 0.93	PJ_DCA_: 1,2,3-TCP→allyl chloride	1.20 ± 0.31 × 10^5^	1.18 ± 0.07 × 10^7^	98.3	1.00 ± 0.06 × 10^7^
	61.39 ± 0.00	PJ_DCA_: 1,2-DCP→propene	1.20 ± 0.31 × 10^5^	9.03 ± 1.69 × 10^6^	75.3	5.80 ± 1.08 × 10^6^
	11.44 ± 1.16^*f*^	PJ_DCA_: 1,1,2,2-TeCA→1,1,2-TCA/TCE/*c*DCE/*t*DCE	1.20 ± 0.31 × 10^5^	4.98 ± 0.33 × 10^6^	41.5	1.70 ± 0.10 × 10^7^
	0	PJ_DCA_: -*^h^*	2.11 ± 0.14 × 10^5^	1.50 ± 0.15 × 10^5^	0.7	NC^*i*^
Strain PJ_TCA_	64.54 ± 0.00	PJ_TCA_: 1,1,2-TCA→VC (8th)	1.02 ± 0.08 × 10^6^	1.24 ± 0.07 × 10^8^	121.6	7.61 ± 0.44 × 10^7^
	58.29 ± 6.25	PJ_TCA_*^j^*: 1,1,2-TCA→VC (2nd)	5.59 ± 1.34 × 10^5^	5.85 ± 0.19 × 10^7^	104.7	3.98 ± 0.12 × 10^7^
	74.84 + 0.02	PJ_TCA_: 1,2-DCA→ETH	1.99 ± 0.38 × 10^5^	2.95 ± 0.51 × 10^7^	148.2	1.57 ± 0.27 × 10^7^
	53.04 ± 0.78	PJ_TCA_: 1,2,3-TCP→allyl chloride	1.99 ± 0.38 × 10^5^	2.80 ± 0.21 × 10^7^	140.7	2.10 ± 0.15 × 10^7^
	60.73 ± 0.01	PJ_TCA_: 1,2-DCP→propene	1.99 ± 0.38 × 10^5^	1.62 ± 0.08 × 10^7^	81.4	1.05 ± 0.05 × 10^7^
	19.98 ± 0.38^*g*^	PJ_TCA_: 1,1,2,2-TeCA→1,1,2-TCA/TCE/*c*DCE/*t*DCE	1.99 ± 0.38 × 10^5^	1.29 ± 0.24 × 10^7^	64.8	2.55 ± 0.47 × 10^7^
	25.73 ± 0.72	PJ_TCA_: 1,2-DBA→ETH	1.39 ± 0.31 × 10^5^	2.02 ± 0.17 × 10^7^	145.3	3.11 ± 0.27 × 10^7^
	0	PJ_TCA_: -*^h^*	1.72 ± 0.06 × 10^5^	1.60 ± 0.04 × 10^5^	0.9	NC^*i*^

^*a*^ The total content of each chlorinated compound consumed in dehalogenation process.

^*b*^ Average and the standard deviations of cell numbers were calculated from the measurements in triplicate cultures.

^*c*^ When a utilizable electron acceptor was completely depleted.

^*d*^ Fold increase in cell densities is reported as the average of the measurements in triplicate cultures.

^*e*^ Growth yields were calculated as increases of cells per μmol Cl^− ^or Br^−^ released.

^*f*^ 11.44 ± 1.16 μmol.

^*g*^ 19.98 ± 0.38 μmol a mixture of *cDCE* and *t*DCE* *was produced via organic-halogen respiration, while TCE is the product of 1,1,2,2-TeCA dehalogenation via abiotic reaction.

^*h*^ -, no chlorinated compound was provided.

^*i*^ NC, yields were not calculated for insignificant growth.

^*j*^ The enrichment culture was incubated with added acetate as carbon source rather than acetate produced by lactate fermention.

### Identification and phylogenetic characterization of novel *Dhc* strains capable of dihaloelimination

**Fig 4:**
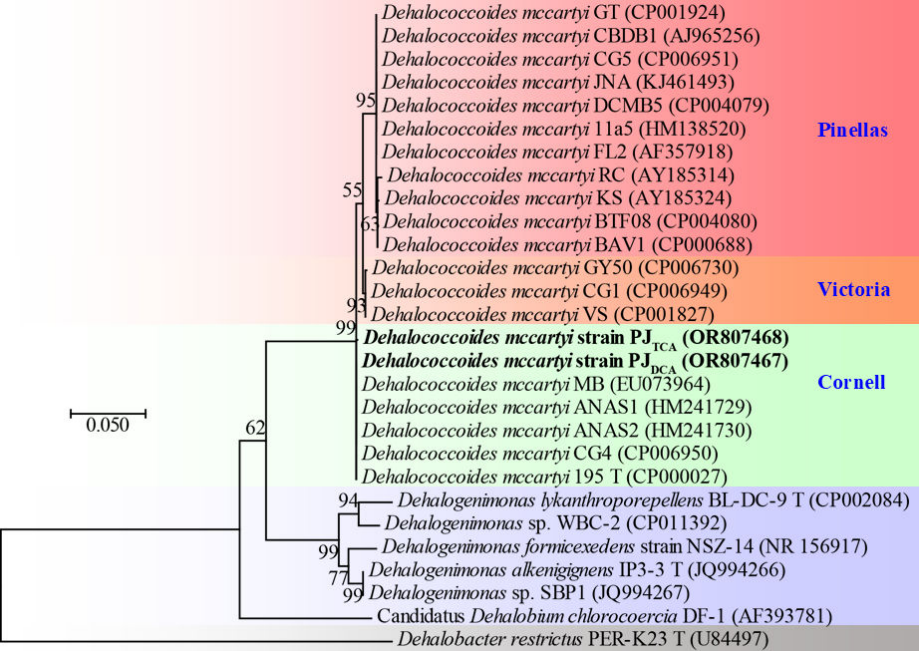
Maximum likelihood tree based on analysis of 16S rRNA gene sequences. It is demonstrated that the phylogenetic affiliation of strains PJ_DCA_ and PJ_TCA_ to species within the phylum *Chloroflexota*. The 16S rRNA gene sequence of *Dhb* strain PER-K23 is included as an outgroup. Provided in parentheses are GenBank accession numbers. Bootstrap support values, expressed as a percentage of 1,000 resamplings, are shown at the branch points. The scale bar indicates the numbers of nucleotide differences per sequence.

Initial screening using *Dhc*-specific primers successfully amplified partial 16S rRNA gene sequences (∼620 bp) from both PJ_DCA_ and PJ_TCA_ enrichment cultures (Fig. S4), while no amplification was observed with *Dhgm*-specific primers. Pairwise comparisons of 16S rRNA gene sequences revealed 98%–100% identity with known *Dhc* strains, confirming the affiliation of these strains with the genus *Dhc*. The combined results of PCR amplification and Sanger sequencing revealed the presence of two novel *Dhc* strains, designated as strains PJ_DCA_ and PJ_TCA_, involved in the dihaloelimination of multiple vicinal halogenated aliphatic alkanes. Metagenomic sequencing enabled the assembly of complete 16S rRNA gene sequences (1,493 bp) of strains PJ_DCA_ and PJ_TCA_. Phylogenetic analysis (Fig. 4) demonstrated that these sequences displayed 99.9% similarity and clustered within the Cornell group, which includes *Dhc* strains such as MB, ANAS1/2, CG4, and 195. Notably, while these reference strains are known to dehalogenate 1,2-DCA, chloroethenes, or polychlorinated biphenyls, they lack the ability to respire on 1,1,2-TCA, highlighting the unique metabolic capabilities of strains PJ_DCA_ and PJ_TCA_. Moreover, the successful amplification of *dcpA*-like gene sequences in both PJ_DCA_ and PJ_TCA_ cultures (Fig. S5) provides evidence that the *Dhc* strains in these enrichments possess the genetic machinery necessary for the observed dehalogenation activity.

### Genomic insights into novel *Dhc* strains with specialized dihaloelimination capability

Metagenome sequencing of the PJ_DCA_ and PJ_TCA_ enrichment cultures yielded 25 and 28 MAGs, respectively, with high-quality genomes predominating (PJ_DCA_: 21 high-quality and 4 medium-quality; PJ_TCA_: 22 high-quality and 6 medium-quality). Of these, only strain PJ_DCA_ and strain PJ_TCA_ harbor reductive dehalogenase (Rdh) gene repertoires, with 29 and 31 full-length reductive dehalogenase catalytic subunit A (*rdhA*) genes, respectively (24 and 18 accompanied by adjacent *rdhB* genes). The draft genomes of PJ_DCA_ and PJ_TCA_ exhibited distinct assembly characteristics. Strain PJ_DCA_ was assembled into a single, circularized 1,572,384 bp contig (48.8% GC content) with exceptional quality metrics (completeness: 99.0%; contamination: 0%). In contrast, the genome (1,524,109 bp, 49.5% GC content) of strain PJ_TCA_ was distributed across 11 contigs (N50 = 777,519 bp) while maintaining comparable completeness (99.0%) despite slightly elevated contamination (2.97%). These two genomes encode 1,719 and 1,612 predicted protein-coding genes, respectively, exhibiting similar genomic characteristics to previously sequenced *Dhc* strains (Table S3). Comparative genomics revealed that strains PJ_DCA_ and PJ_TCA_ share 97.7%–98.5% average nucleotide identity (ANI) and 79.9%–87.4% digital DNA-DNA hybridization (dDDH) identity with *Dhc* strains MB, CG4, and 195 from the Cornell group, exceeding the established thresholds for species demarcation (95% ANI and 70% dDDH) (32, 33) (Table S4).

**Fig 5:**
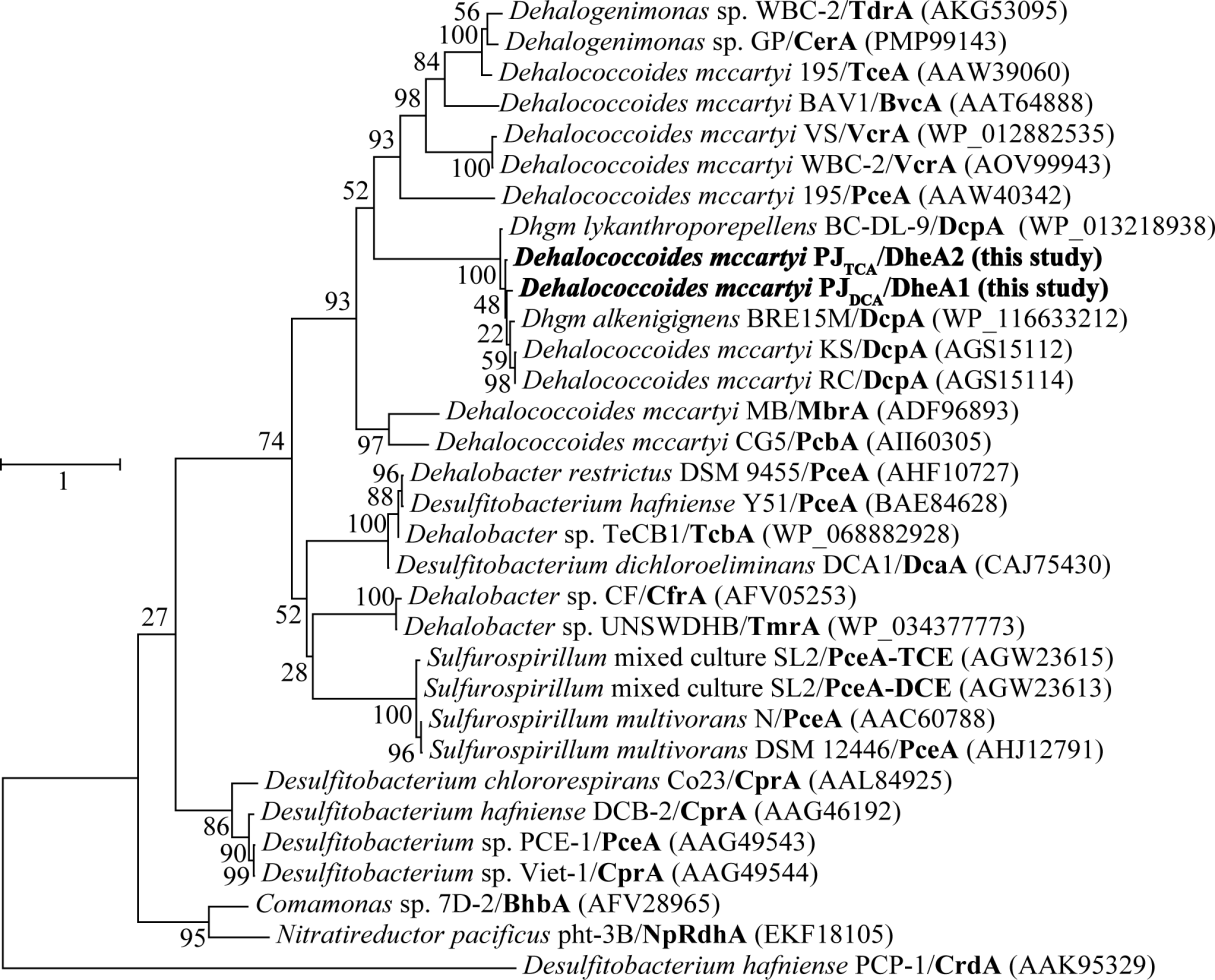
Phylogenetic relationship of DcpA in strains PJ_DCA_ and PJ_TCA_ with 30 characterized reductive dehalogenases. Detailed information on dehalogenation substrates, products, and the corresponding methods of function identification for the 30 reductive dehalogenases can be found in Table S8. Numbers at the branch points represent bootstrap percentages calculated from 1,000 replicate trees. The scale bar represents the amino acid sequence divergence. GenBank accession numbers are provided in parentheses.

Notably, a 1,455-bp homolog of *dcpA* gene, responsible for the dihaloelimination of vicinal halogenated C_2_−C_3_ alkanes (e.g., 1,2-DCA, 1,1,2-TCA, 1,2-DCP, and 1,2,3-TCP) (34, 35), was identified in both strains PJ_DCA_ and PJ_TCA_, designated as *dheA* (originated from dihaloelimination). Pairwise comparison showed that DheA1 (strain PJ_DCA_) and DheA2 (strain PJ_TCA_) share a 93.2% amino acid similarity. Phylogenetic analysis of RDases revealed that DheA1 and DheA2 cluster with DcpA homologs from *Dhc* strains KS and RC (35), as well as *Dhgm* strain BL-DC-9^9, 35^ (Fig. 5). DheA1 of strain PJ_DCA_ exhibits 93.1% amino acid sequence similarity to DcpA from *Dhc* strains KS and RC, while DheA2 of strain PJ_TCA_ shares 94.8% similarity with DcpA from *Dhgm* strain BL-DC-9. Multiple sequence alignment uncovered significant amino acid distinctions between DcpA homologs from *Dhc* strains RC/KS/PJ_DCA_ and those from *Dhgm* strain BL-DC-9/*Dhc* strain PJ_TCA_ (Fig. S6), suggesting potential functional divergence in their catalytic mechanisms. Moreover, the genomes of strains PJ_DCA_ and PJ_TCA_ encode complete OHR-complex machinery, including Type II CISM proteins (OmeA/B) and hydrogenase components (HupL/S/X) and DheA, suggesting functional electron transport chains capable of supporting organohalide respiration.

### Proteomics reveals key reductive dehalogenases in *Dhc*-mediated dihaloelimination

**Fig 6:**
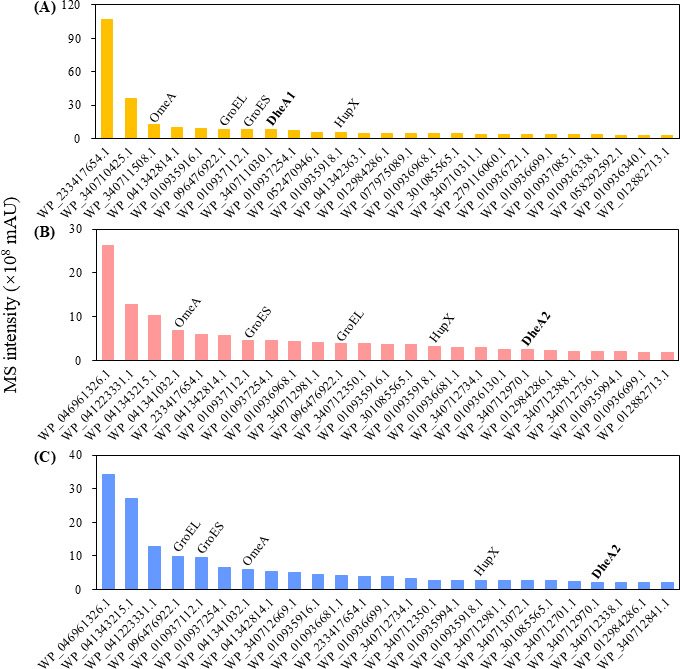
Protein profiling of *Dhc* strains grown with 1,2-DCA, 1,2-DCP, and 1,1,2-TCA. (**A**) shows top 25 expressed proteins identified in strain PJ_DCA_ from 1,2-DCA-dehalogenating culture. (**B and C**) show top 25 expressed proteins identified in strain PJ_TCA_ from 1,2-DCP- and 1,1,2-TCA-dehalogenating PJ_TCA_ cultures, respectively. See Table S5 for the complete list of identified proteins and their expression profiles in strain PJ_DCA_ or PJ_TCA_ grown with 1,2-DCA, 1,2-DCP, and 1,1,2-TCA. Reductive dehalogenases are indicated in bold.

Proteomic analysis of *Dhc* strains across three distinct dehalogenating cultures (grown with 1,2-DCA, 1,1,2-TCA, and 1,2-DCP) identified 761, 828, and 721 proteins, respectively (Table S5). Notably, two DcpA-like RdhA dominated the proteomes: DheA1 (WP_340711030) in strain PJ_DCA_ from the 1,2-DCA-dehalogenating culture (Fig. 6A) and DheA2 (WP_340712970) in strain PJ_TCA_ from both the 1,2-DCP- and 1,1,2-TCA-dehalogenating culture (Fig. 6B and C). Despite their functional importance, the corresponding membrane-anchoring RdhB was not detected, consistent with reported challenges in solubilizing and detecting integral membrane RdhB proteins via mass spectrometry in *Dhc* proteomics (36). In addition to DheA, two other RdhA (WP_010937203 and WP_340710341) were expressed in the 1,2-DCA-dehalogenating PJ_DCA_ culture. In contrast, the 1,1,2-TCA-dehalogenating PJ_TCA_ culture expressed eight RdhA (WP_340713280, WP_278812923, WP_278813062, WP_077974976, WP_010937203, WP_340712352, WP_340712476, WP_340713276), while the 1,2-DCP-dehalogenating PJ_TCA_ culture expressed six RdhA (WP_278812923, WP_077974976, WP_010937203, WP_340712352, WP_340713276, WP_278813062). Phylogenetic analysis revealed that these RdhA shared less than 40% amino acid identity with characterized RdhA (Fig. S7), suggesting their potential functional novelty. Strikingly, DheA emerged as the predominant dehalogenase across all cultures, consistently ranking among the top 22 most abundant proteins (8th, 20nd, and 19th positions in 1,2-DCA-, 1,1,2-TCA-, and 1,2-DCP-dehalogenating cultures, respectively). In contrast, other RdhA showed significantly lower expression levels (abundance rankings >200), strongly implicating DheA1 and DheA2 as the primary catalysts for dihaloelimination of vicinal halogenated C_2_-C_3_ alkanes, including 1,2-DCA, 1,1,2-TCA, 1,2-DCP. The proteomes also revealed high expression level of OHR complex components, including OmeA and HupX proteins, confirming functional electron transport chains supporting organohalide respiration. Elevated expression of molecular chaperones (GroES/EL, DnaK) suggests *Dhc* strains employ sophisticated stress response systems to mitigate toxicity from 1,2-DCA, 1,2-DCP, or 1,1,2-TCA.

## DISCUSSION

### Discovery of novel *Dhc* strains capable of dihaloeliminating multiple vicinal halogenated C_2_−C_3_ alkanes

In this study, we successfully obtained two *Dhc*-containing enrichment cultures, designated as PJ_DCA_ and PJ_TCA_, from petroleum-contaminated soil in Northeast China. Given that petroleum hydrocarbon-impacted sites are frequently co-contaminated with halogenated solvents, this origin provides a plausible ecological basis for the enrichment of microbial populations specialized in dehalogenating chlorinated organic compounds (37). The obtained cultures exhibit remarkable dihaloelimination capabilities toward a broad spectrum of vicinal halogenated C_2_−C_3_ alkanes, including 1,2-DCA, 1,1,2-TCA, 1,2-DCP, 1,2,3-TCP, 1,1,2,2-TeCA, and 1,2-DBA. The robust growth of *Dhc* and the absence of known 1,2-DCA or 1,1,2-TCA dechlorinators, such as *Dhb*, *Dhgm*, *Trichlorobacter*, and *Desulfitobacterium*, underscores the pivotal role of *Dhc* strains PJ_DCA_ and PJ_TCA_ in driving dihaloelimination reactions. This finding substantially expands the known substrate range of *Dhc* (Table S6), as previous reports were confined to the dihaloelimination of a narrow set of haloalkanes (i.e., 1,2-DCA, 1,2-DCP, and 1,2-DBA). While certain *Dhc* strains (195, BAV1, 11a, VS) have been reported to dehalogenate 1,2-DCA to ethene, they lack the ability to metabolize 1,1,2-TCA (14, 17, 18). Similarly, *Dhc* strains RC and KS, which harbor the *dcpA* gene, are known to dihaloeliminate 1,2-DCP to propene, but their capabilities toward other chlorinated substrates remain unexplored (35, 38). A recent study reported the dihaloelimination of 1,2-DBA to ethene in a *Dhc*-containing enrichment culture (39). However, no *Dhc* strains capable of dehalogenating other haloalkanes, such as 1,1,2-TCA, 1,2,3-TCP, and 1,1,2,2-TeCA, had been identified. Our study represents the first comprehensive demonstration of *Dhc*-mediated dihaloelimination of multiple vicinal halogenated aliphatic alkanes, including 1,1,2-TCA, 1,2,3-TCP, 1,1,2,2-TeCA, 1,2-DCA, 1,2-DCP, and 1,2-DBA. Notably, strains PJ_DCA_ and PJ_TCA_ represent the first *Dhc* strains capable of dehalogenating 1,1,2-TCA to VC, 1,2,3-TCP to allyl chloride, and 1,1,2,2-TeCA to *c*DCE/*t*DCE. This discovery significantly expands the known metabolic diversity of *Dhc* and highlights their potential for bioremediation of a broader range of chlorinated alkanes.

Phylogenetic analysis based on 16S rRNA gene sequences revealed that strains PJ_DCA_ and PJ_TCA_ cluster within the Cornell group, exhibiting 99.9%–100% similarity to *Dhc* strains MB, 195, CG4, ANAS1, and ANAS2, which are incapable of dehalogenating both 1,2-DCA and 1,1,2-TCA. Comparative genomic analysis further confirmed that the draft genomes of strains PJ_DCA_ and PJ_TCA_ share the closest relationship with strains CG4, MB, and 195 within the Cornell group, as evidenced by ANI and dDDH values. In contrast, these values fall below the threshold for strain GY50 (40) (Victoria group) and strains KS/RC (35, 38) (Pinellas group), reinforcing the novelty of strains PJ_DCA_ and PJ_TCA_. These findings provide valuable insights into the biogeography and evolutionary dynamics of *Dhc*, suggesting that functional diversification may have occurred.

**Fig 7:**
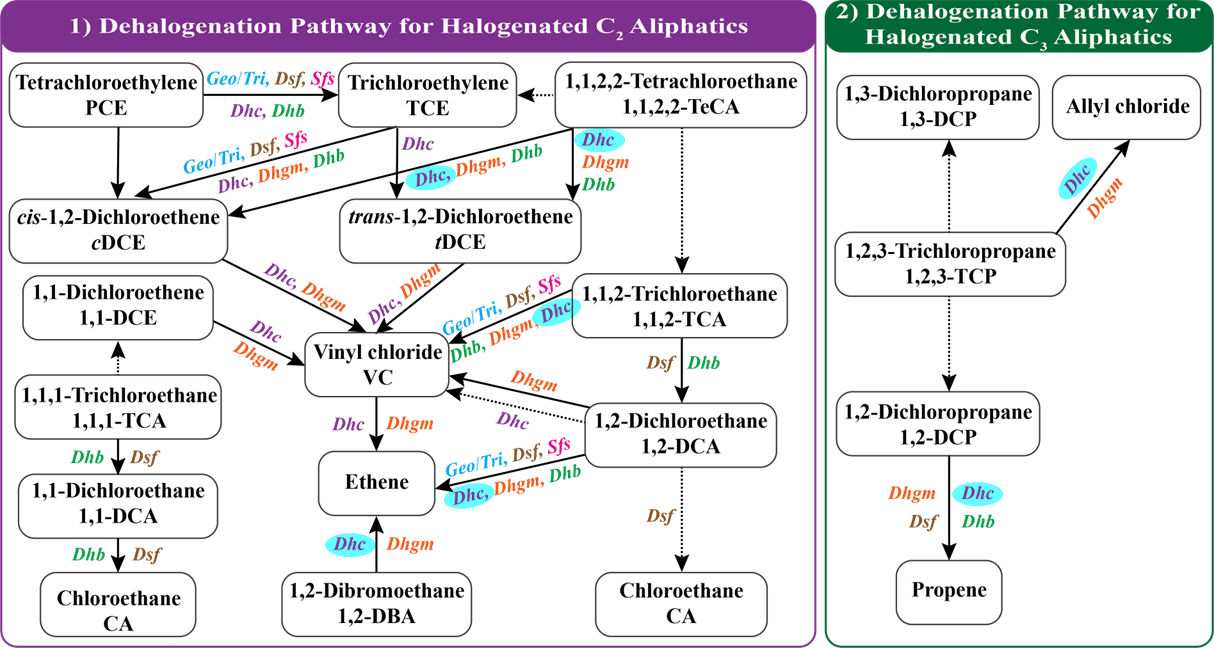
Dehalogenation pathways of selected halogenated organic compounds and associated OHRB. This schematic consolidates the novel findings from this study (highlighted in cyan) with previously reported dehalogenation pathways and their associated OHRB. Note that metabolic capabilities are highly strain-specific; therefore, individual steps within a pathway are often catalyzed by specialized strains rather than all members of the indicated genus (41). Dashed lines denote abiotic reactions or biological transformations that do not support growth. *Dhc*, *Dehalococcoides*; *Dhgm*, *Dehalogenimonas*; *Dhb*, *Dehalobacter*; *Dsf*, *Desulfitobacterium*; *Sfs*, *Sulfurospirillum*; *Geo/Tri*, *Geobacter*/*Trichlorobacter* lineages.

The discovery of strains PJ_DCA_ and PJ_TCA_ has significant implications for understanding the environmental fate and longevity of haloalkanes at contaminated sites. By integrating our findings with established knowledge, Fig. 7 presents a comprehensive overview of microbial transformation pathways for haloalkanes under anaerobic conditions, including the newly identified pathways mediated by strains PJ_DCA_ and PJ_TCA_. By mapping the stepwise degradation of these compounds and identifying the key microbial players, this work provides critical insights into the mechanisms governing the natural attenuation of chlorinated pollutants in anoxic environments. Such knowledge is essential for predicting the long-term behavior of these contaminants and for informing the development of targeted bioremediation strategies to mitigate their environmental impact.

### Genetic and functional divergence in dihaloelimination-capable RDases

Dihaloelimination is a thermodynamically favorable and critical detoxification pathway for vicinal halogenated aliphatic alkanes such as 1,2-DCA and 1,1,2-TCA (6). Based on standard Gibbs free energy calculations, dihaloelimination yields substantially more free energy than alternative degradation pathways. For example, the dihaloelimination of 1,2-DCA to ethene (Δ*G*⁰′ ≈ –187.9 kJ/reaction) is considerably more exergonic than its hydrogenolysis to chloroethane (ca. –135.0 kJ/reaction) (42). This thermodynamic advantage becomes even more pronounced for highly chlorinated ethanes, such as 1,1,2-TCA and 1,1,2,2-TeCA, where dihaloelimination consistently releases 40–60 kJ/mol more energy than hydrogenolysis. Such a significant energetic advantage provides a compelling explanation for why *Dhc* strains like PJ_DCA_ and PJ_TCA_ thrive via dihaloelimination in anoxic environments; it allows them to maximize energy conservation per mole of substrate and gain a competitive edge in electron acceptor-limited niches. Despite this strong evolutionary and energetic incentive, the repertoire of RDases conclusively shown to catalyze reductive dihaloelimination remains limited (Table S7). These enzymes exhibit significant functional diversification despite sharing conserved core domains (N-terminal twin-arginine motif and C-terminal iron–sulfur cluster-binding motifs) (Fig. S6). For instance, DcpA from *Dhgm* strains BL-DC-9, BRE15M, IP3-3, and SPB-1 exhibits a broad dihaloelimination range (e.g., 1,2-DCA, 1,1,2-TCA, 1,2-DCP, 1,2,3-TCP, 1,1,2,2-TeCA, and 1,2-DBA) (9, 11, 43). While DcaA from *Desulfitobacterium dichloroeliminans* DCA1 and *Dehalobacter* strain WL can also dehalogenate multiple vicinal halogenated aliphatic alkanes via dihaloelimination in addition to transforming chloroethenes (PCE, TCE) via hydrogenolysis (7, 8). Similarly, TceA from *Dhc* strain 195, BvcA from *Dhc* strain BAV1, and VcrA from *Dhc* strains VS and 11a, while capable of hydrogenolytic dehalogenation of chloroethenes (e.g., TCE, *c*DCE, 1,1-DCE) and dihaloelimination of 1,2-DCA, show no activity toward 1,1,2-TCA (14, 44–46). These observed divergences underscore a fundamental challenge: global sequence alignment is insufficient for predicting RDase function, a limitation that is most starkly revealed within closely related ortholog groups. The chloroalkane-reducing ortholog group OG 97 serves as a compelling paradigm for this principle. Within this group, enzymes sharing high sequence identity, such as CfrA and DcrA (>95%), exhibit starkly divergent substrate preferences and catalytic outcomes for 1,1,2-TCA: CfrA primarily dehalogenates CF and 1,1,1-TCA via hydrogenolysis, converting 1,1,2-TCA to 1,2-DCA, whereas DcrA favors 1,1-DCA and transforms 1,1,2-TCA almost exclusively to VC via dihaloelimination (20). This dramatic shift in both substrate preference and reaction mechanism was experimentally pinpointed by Picott et al. (47) who demonstrated that introducing only two mutations (Y80W and F125W) into CfrA was sufficient to confer DcrA-like activity toward 1,1-DCA and redirect 1,1,2-TCA transformation toward dihaloelimination to VC.

Extending this principle to the enzymes identified in our study, comparative sequence analysis (Fig. S6) reveals that DheA, while harboring the conserved twin-arginine translocation (TAT) signal, [4Fe–4S] cluster-binding motifs, and catalytic core domains essential for reductive dehalogenation, lacks the specific aromatic residues (corresponding to Y80 and F125) that function as the mechanistic switch in OG 97. This observation suggests that the dihaloelimination capacity of DheA arises from a distinct structural configuration rather than a universally conserved switch motif. Notably, RDases such as DcpA from *Dehalogenimonas* and DcaA from *Desulfitobacterium* exhibit limited sequence identity, yet both mediate dihaloelimination of vicinal haloalkanes, supporting the hypothesis of convergent evolution of this enzymatic function (7–9). In contrast, canonical hydrogenolysis-catalyzing enzymes (mono-dehalogenases), such as typical PceA homologs, likely possess rigid active site constraints that either restrict simultaneous interaction with two halogens or thermodynamically favor single electron transfer and protonation events.

Similarly, DcaA share 88.2%–91.5% sequence similarity with PceA from *Dhb* strain PER-K23, yet their substrate profiles differ substantially. These cases underscore that profound functional divergence can arise from subtle, localized sequence variations not captured by global alignment, a critical consideration when interpreting the sequence-function relationships of the DheA enzymes characterized in this study. Structural and mutagenesis studies in other RDase families have identified analogous critical control points. Mutation of Tyr298 to Phe abolishes PCE dehalogenation activity in both PceA from *Desulfitobacterium hafniense* Y51 and DcaA from *Desulfitobacterium dichloroeliminans* DCA1 (48). Likewise, DcaA variants W118F, W432F, and T294V exhibit impaired function and altered substrate specificity, suggesting that these residues (W118, W432, and T294) are key components of the DcaA active site. In NpRdhA, the A419M variant shifts substrate preference from di- to mono-halogenated phenols, while the K488Q mutation abolishes reductase activity (49). In our study, the accelerated dehalogenation kinetics in strain PJ_TCA_ (Fig. 2), together with subtle amino acid differences between DheA1 and DheA2, suggest that these enzymes have undergone substrate-specific adaptations to 1,2-DCA and 1,1,2-TCA, respectively. Future structural modeling of DheA—alongside comparison with RDases from versatile genera like *Trichlorobacter*/*Geobacter* (e.g., strain IAE) and with well-characterized OG 97 family enzymes—will be essential to define the steric and electrostatic determinants that support dihaloelimination across divergent RDase lineages. Such insights will require integrated approaches, including comparative genomics, heterologous expression, and site-directed mutagenesis, to map substrate recognition elements and catalytic residues responsible for the distinct reactivity of dihaloeliminating enzymes.

### Implications for bioremediation applications

Over the past decade, OHRB-based bioremediation has emerged as a cornerstone technology for the remediation of chlorinated solvent-contaminated sites (50). Significant advancements in anaerobic treatment have facilitated the development and commercialization of *Dhc*-containing bioaugmentation cultures, which have proven particularly effective at sites where native microbial populations fail to support rapid or complete reductive dehalogenation. A prominent example is the commercial KB-1 consortium, which has been successfully implemented for *in situ* bioremediation (51). In such applications, *Dhc* strains are consistently maintained as the dominant dechlorinators of PCE, TCE, *c*DCE, and VC, ultimately yielding non-toxic ethene as the final product. This study reports the discovery of two novel *Dhc* strains, PJ_DCA_ and PJ_TCA_, which exhibit unique dihaloelimination capabilities toward multiple halogenated alkanes. Critically, within the genus *Dhc*, the enzymatic mechanism of dihaloelimination remains less explored compared to classical monodehalogenation. Our integrated meta-omics approach provides the functional and genomic evidence linking these strains and a specific reductive dehalogenase gene (*dheA*) to this activity, establishing a foundation for future mechanistic characterization. These findings significantly expanded our understanding of the metabolic diversity within the *Dhc* genus and highlight their potential for remediating sites co-contaminated with halogenated alkanes through dihaloelimination pathways. However, a key consideration for application is that the end products of 1,1,2-TCA and 1,1,2,2-TeCA dihaloelimination include VC and DCEs (i.e., *c*DCE and *t*DCE), which are persistent toxic pollutants. To achieve complete detoxification, future research will focus on (i) the isolation and detailed physiological characterization of strains PJ_DCA_ and PJ_TCA_, (ii) the development of synergistic microbial consortia that combine these dihaloeliminating strains with established VC- and DCE-dehalogenating *Dhc* strains, and (iii) evaluating the dehalogenation kinetics and microbial interactions in scenarios with mixtures of halogenated pollutants, such as co-contamination with 1,1,2-TCA and 1,2-DCA or mixtures of chloroethanes with chloroethenes (e.g., 1,2-DCA and VC), as commonly encountered at co-contaminated sites, to better predict and optimize *in situ* bioremediation performance. Such integrated strategies aim to funnel pollutants completely to ethene, thereby enhancing the efficacy and sustainability of bioremediation strategies for complex halogenated contaminant mixtures.

## Data Availability

All sequencing data generated in this study have been deposited in publicly accessible repositories to ensure data transparency and reproducibility. The amplicon sequencing data are available in the Sequence Read Archive (SRA) with the accession number SRR26488200. Raw metagenome sequences derived from soil samples and the PJ_DCA_ and PJ_TCA_ enrichment cultures have been deposited in the SRA under accession numbers SRR26483806, SRR26484130, and SRR26484016, respectively. The draft genome assemblies of strains PJ_DCA_ and PJ_TCA_ have been submitted to GenBank and are accessible under the accession numbers JAWQJB000000000 and JAWQJC000000000, respectively. Additionally, the 16S rRNA gene sequences of strains PJ_DCA_ and PJ_TCA_ are available in GenBank under accession numbers OR807467 and OR807468, respectively. The *dheA1* and *dheA2* gene sequences from strains PJ_DCA_ and PJ_TCA_ have also been deposited in GenBank under accession numbers OR820144 and OR820145, respectively. All data are freely accessible for further research and analysis. See the supplemental material for the details of DNA extraction, PCR, Sanger sequencing, amplicon sequencing, qPCR, and phylogenetic analysis.

## SUPPLEMENTAL MATERIAL

Supplemental material aem.00209-26-s0001.docx Supplemental methods, Tables S1 to S9, and Fig. S1 to S7.

Table S5 aem.00209-26-s0002.xlsx Results of proteomic analysis.

ASM does not own the copyrights to Supplemental Material that may be linked to, or accessed through, an article. The authors have granted ASM a non-exclusive, world-wide license to publish the Supplemental Material files. Please contact the corresponding author directly for reuse.
