# Supplementary material for: Revisiting the dihaloelimination potential of Dehalococcoides revealed by genomic and proteomic analyses
Source: Appl Environ Microbiol. 2026 May 7;92(6):e00209-26. doi: 10.1128/aem.00209-26 (PMC13274352; doi:10.1128/aem.00209-26)
Supplement: Supplemental material — Supplemental methods, Tables S1 to S9, and Fig. S1 to S7. [file aem.00209-26-s0001.docx]

**Revisiting the Dihaloelimination Potential of *Dehalococcoides* Revealed by Genomic and Proteomic Analyses**

Xiaocui Li^1,2^, Hongming Cai^3^, Hongyan Wang^1,2^, Jiubin Chen^3^, Tong Zhang^4^, Haiwei Wei^2^, Lian Yu^5^, Zongming Xiu^6^, Jun Yan^2^, Yi Yang^1,2,7,8*^

^1^Key Laboratory of Forest Ecology and Silviculture, Institute of Applied Ecology, Chinese Academy of Sciences, Shenyang, Liaoning, 110016, China;

^2^Key Laboratory of Pollution Ecology and Environmental Engineering, Institute of Applied Ecology, Chinese Academy of Sciences; Shenyang, Liaoning 110016, China;

^3^School of Earth System Science, Tianjin University, Tianjin, 300350, China;

^4^ College of Environmental Science and Engineering, Nankai University, Tianjin, 300350, China

^5^Department of Environmental Engineering, Beijing Institute of Petrochemical Technology, Beijing, 102617, China;

^6^Zhejiang Key Laboratory of Environment and Health of New Pollutants, School of Environment, Hangzhou Institute for Advanced Study, University of Chinese Academy of Sciences, Hangzhou, China;

^7^Shanghai Key Laboratory of Polar Life and Environment Sciences, Shanghai Jiao Tong University, Shanghai, 200030, China;

^8^Key Laboratory of Polar Ecosystem and Climate Change, Shanghai Jiao Tong University, Shanghai, 200030, China.

*** Corresponding authors**

Yi Yang, Institute of Applied Ecology, Chinese Academy of Sciences, 504 South Building, 72 Wenhua Road, Shenyang, Liaoning 110016, China, Phone: +86-2483970426, E-mail: yangyi@iae.ac.cn

**Supplementary materials**

**Materials and Methods**

**DNA extraction and 16S rRNA gene amplicon sequencing.** Following complete dechlorination of either 1,2-DCA or 1,1,2-TCA, microbial cells were harvested from 1 mL culture suspensions through vacuum filtration onto 0.22 μm mixed cellulose ester (MCE) filters (JINTENG, Tianjin, China). Genomic DNA was extracted using the TIANamp Soil DNA Kit (TIANGEN Inc., Beijing, China) in strict accordance with the manufacturer’s protocol. DNA concentrations and purity were quantified using a Qubit 3.0 fluorometer (Invitrogen, Carlsbad, CA, USA). The V3−V4 hypervariable regions of bacterial and archaeal 16S rRNA genes were amplified using the universal primer sets V3-V4-F and V3-V4-R (**Table S9**). Sequencing libraries were prepared using the MetaVx™ Library construction Kit (GENEWIZ, USA) following the manufacturer's instructions. Paired-end sequencing targeting 16S rRNA genes was conducted on an Illumina MiSeq PE250 platform (Illumina Inc., San Diego, CA, USA) following established protocols.^1^ Raw sequence data were processed using a rigorous bioinformatics pipeline. Sequences containing ambiguous nucleotides (“N”) or those shorter than 200 bp were removed using Cutadapt version 1.9.1. Chimeric sequences were identified and eliminated using VSEARCH version 1.9.6 implemented in Qiime version 1.9.1.^2-4^ Quality-filtered sequences were clustered into operational taxonomic units (OTUs) at a 97% sequence similarity threshold using the UPARSE algorithm.

**PCR and Sanger sequencing. PCR** amplification targeting 16S rRNA genes of *Dhc* and *Dhgm*, along with the vicinal dihaloelimination-associated RDase gene *dcpA*, was performed using a Veriti 96-well thermal cycler (Thermo Fisher Scientific, Waltham, MA, USA). Published primer sets (**Table S8**) were employed, along with optimized PCR mixture system and thermocycler program as described previously.^5^ Amplification products were separated via 1.5% agarose gel electrophoresis and visualized using GelRed staining. Sanger sequencing was performed on an Applied Biosystems 3730XL sequencer (Thermo Fisher Scientific).^6^

**Quantitative real-time PCR (qPCR) assay.** Real-time quantification of *Dhc* 16S rRNA gene copies during dihaloelimination of halogenated alkanes was performed using TaqMan probe**-**based qPCR (Applied Biosystems QuantStudio 3, Waltham, MA, USA), adhering to the procedures and detailed recipes outlined in the published protocol.^5^ The assay employed the published primer set *Dhc*1200F and *Dhc*1271R, along with the TaqMan probe *Dhc*1240probe (**Table S8**). Calibration curves were generated using three independent dilution series of the plasmid DNA carrying a fragment of *Dhc* 16S rRNA gene. The *Dhc* qPCR had an amplification efficiency of 96% and a linear dynamic range of 2.80 × 10^2^ to 2.80 × 10^9^ gene copies per reaction tube. Cell number calculations were based on the assumption of one 16S rRNA gene copy per *Dhc* genome.^7^

**Phylogenetic analysis.** Selected *Dhc*, *Dhgm*, and Candidatus *Dehalobium* 16S rRNA gene sequences were retrieved from the GenBank database and aligned using MEGA version 7.0.21. Protein sequences of 30 characterized RDases (**Table S6**) were retrieved from the Reductive Dehalogenase Database (http://rdasedb.biozone.utoronto.ca) or GenBank. **Table S6** provides information on host OHRB, substrates, corresponding products, and the identification method of these RDases, along with their assigned functions. Phylogenetic analysis of both 16S rRNA gene sequences and (putative) RDase proteins were performed using the Neighbor-Joining method with MEGA v7.0.21 software.^8^ *Dehalobacter restrictus* PER-K23 was selected as an outgroup for the 16S rRNA gene phylogenetic tree. The reliability of the generated trees was assessed by bootstrapping with 1000 replicates.

**Table S1.** Summary of known OHRB capable of dechlorinating 1,2-DCA and/or 1,1,2-TCA.

| **Electron**  **acceptor** | **OHRB** | **End products** | **Functional RDase^b^** | **References** |
| --- | --- | --- | --- | --- |
|  |  |  |  |  |
| **1,2-DCA** | *Dehalococcoides mccartyi* strain 195 | Ethene, VC ^a^ | TceA (AAW39060) | ^9, 10^ |
|  | *Dehalococcoides mccartyi* strain BAV1 | Ethene, VC ^a^ | BvcA (AAT64888) | ^11, 12^ |
|  | *Dehalococcoides mccartyi* strain 11a | Ethene | VcrA (HM138519) | ^13^ |
|  | *Dehalococcoides mccartyi* strain VS | Ethene | VcrA (WP_012882535) | ^14, 15^ |
|  | *Dehalogenimonas lykanthroporepellens* strain BL-DC-9 | Ethene | DcpA (WP_013218938) | ^16, 17^ |
|  | *Dehalogenimonas alkenigignens* strains IP3-3, SPB-1 | Ethene | DcpA (^NA^) | ^18^ |
|  | *Dehalogenimonas alkenigignens* strain BRE15M | Ethene | DcpA | ^19^ |
|  | *Dehalogenimonas etheniformans* strain GP | Ethene | ^NA^ | ^20^ |
|  | *Desulfitobacterium dichloroeliminans* strain DCA1 | Ethene | DcaA (CAJ75430.1) | ^21-23^ |
|  | *Desulfitobacterium* sp. strain AusDCA | Ethene | DcaA (AFV09851.1) | ^23^ |
|  | *Dehalobacter* sp. strain WL | Ethene | RdhA1 (ACH87594.1) | ^24^ |
|  | *Trichlorobacter* sp. strains AY, IAE | Ethene | ^NA^ | ^5, 25^ |
| **1,1,2-TCA** | *Dehalogenimonas lykanthroporepellens* strain BL-DC-9 | VC | DcpA (WP_013218938) | ^16, 17^ |
|  | *Dehalogenimonas alkenigignens* strains IP3-3, SPB-1 | VC | DcpA (^NA^) | ^18^ |
|  | *Dehalogenimonas alkenigignens* strain BRE15M | VC | DcpA | ^19^ |
|  | *Desulfitobacterium dichloroeliminans* strain DCA1 | VC | DcaA (CAJ75430.1) | ^21-23^ |
|  | *Desulfitobacterium* sp. strain PR | 1,2-DCA, CA, VC ^a^, Ethene ^a^ | ^NA^ | ^26^ |
|  | *Dehalobacter restrictus* strains CF, DCA, 8M | 1,2-DCA, VC | ^NA^ | ^27, 28^ |
|  | *Dehalobacter restrictus* strain UNSWDHB | 1,2-DCA, VC | TmrA (WP_034377773) | ^29^ |
|  | *Dehalobacter* sp. strain WL | VC | ^NA^ | ^24^ |
|  | *Trichlorobacter* sp. strain IAE | VC | ^NA^ | ^5^ |

^a^ Detected in trace amount. ^b^ RDase responsible for 1,2-DCA and/or 1,1,2-TCA dechlorination. ^NA^ 1,2-DCA- and 1,1,2-TCA-dechlorinating RDase are unpublished or unidentified. The NCBI accession number is in parentheses.

**Table S2.** Phylum-level abundances based on 16S rRNA gene amplicon sequences obtained from the soil microcosms and enrichment cultures.

| **Phylum** | **Percentage of the total amplicon sequences (%)** | | | |
| --- | --- | --- | --- | --- |
|  | **1,2-DCA dechlorinating** | | **1,1,2-TCA dechlorinating** | |
|  | **Sediment microcosm** | **PJ_DCA_*^a^*** | **Sediment microcosm** | **PJ_TCA_*^b^*** |
| *Bacillota* | 31.64 | 44.85 | 36.81 | 22.73 |
| *Bacteroidota* | 39.8 | 44.14 | 27.35 | 58.04 |
| WPS-2 | 0.42 | 0.69 | 0.47 | 8.69 |
| *Desulfobacterota* | 1.44 | 0.26 | 2.13 | 1.95 |
| *Cloacimonadota* | 2.28 | 4.39 | 1.5 | 2.14 |
| *Synergistota* | 3.89 | 1.77 | 6.99 | 3.36 |
| *Pseudomonadota* | 8.17 | 0.01 | 7.67 | 0.17 |
| *Chloroflexota* | 4.65 | 0.3 | 9.79 | 0.58 |
| *Spirochaetota* | 0.87 | 0.97 | 0.79 | 1.61 |
| *Elusimicrobiota* | 0 | 0.72 | 0 | 0 |
| *Caldisericota* | 0.48 | 0.54 | 1.09 | 0.59 |
| *Halobacterota* | 1.93 | 0.35 | 0.66 | 0 |
| *Bacteria_*Unclassified | 0.06 | 0.99 | 0.12 | 0.08 |
| *Actinobacteriota* | 2.45 | 0.01 | 2.9 | 0.01 |
| *Thermotogota* | 0.21 | 0.02 | 0.87 | 0.01 |
| *Patescibacteria* | 0.38 | 0 | 0.24 | 0 |
| *Acidobacteriota* | 0.67 | 0 | 0.12 | 0 |
| *Deinococcota* | 0.29 | 0 | 0.1 | 0 |
| *Latescibacterota* | 0.03 | 0 | 0.08 | 0 |
| *Euryarchaeota* | 0 | 0 | 0 | 0 |
| *Armatimonadota* | 0.27 | 0 | 0 | 0 |
| *Edwardsbacteria* | 0.01 | 0 | 0.01 | 0 |
| *Nitrospirota* | 0 | 0 | 0.02 | 0.04 |
| *Hydrogenedentes* | 0.02 | 0 | 0.14 | 0 |
| *Caldatribacteriota* | 0.02 | 0 | 0.09 | 0 |
| *Verrucomicrobiota* | 0 | 0.01 | 0.01 | 0 |
| *Bdellovibrionota* | 0.03 | 0 | 0.03 | 0 |
| WS4 | 0.01 | 0 | 0.02 | 0 |

*^a^* DNA sample was extracted from the fourteenth-generation PJ_DCA_ enrichment culture amended with lactate.

*^b^* DNA sample was extracted from the eleventh-generation PJ_TCA_ enrichment culture amended with lactate.

**Table S3.** Genomic characteristics of selected *Dhc* strains.

| ***Dhc* strain** | **PJ-1** | **PJ-2** | **KS** | **RC** | **195** | **CG4** | **MB** | **VS** | **CG1** |
| --- | --- | --- | --- | --- | --- | --- | --- | --- | --- |
| Genome size (Mbp) | 1.57 | 1.52 | 1.50 | 1.49 | 1.47 | 1.38 | 1.57 | 1.41 | 1.49 |
| G+C content (%) | 48.76 | 49.52 | 47.23 | 47.17 | 48.90 | 48.70 | 48.30 | 47.30 | 46.90 |
| Protein-coding genes | 1719 | 1612 | 1653 | 1671 | 1510 | 1399 | 1643 | 1439 | 1531 |
| rRNA(5S,16S,23S) | 3 | 3 | 3 | 3 | 3 | 3 | 3 | 3 | 3 |
| tRNA | 44 | 43 | 46 | 46 | 46 | 47 | 47 | 47 | 49 |
| *rdhA* gene | 29 | 31 | 31 | 38 | 17 | 15 | 34 | 36 | 35 |
| Cobalamin biosynthetic pathway | **-** | **-** | - | - | - | - | - | - | - |

**Table S4**. Average Nucleotide Identity (ANI) and digital DNA-DNA hybridization (dDDH) between *Dhc* strains PJ_DCA_ or PJ_TCA_ and other *Dhc* strains.

| ***D. mccartyi* strains** | **G+C**  **content (%)** | ANI **(%) and** dDDH **(%)** | | | | | | | |
| --- | --- | --- | --- | --- | --- | --- | --- | --- | --- |
|  |  | **1** | **2** | **3** | **4** | **5** | **6** | **7** | **8** |
| **1. *D. mccartyi* PJ_DCA_** | **48.76** |  | **83.6** | **87.4** | **81.3** | **82.0** | **38.8** | **29.5** | **29.5** |
| **2. *D. mccartyi* PJ_TCA_** | **49.52** | **97.99** |  | **82.6** | **79.9** | **82.3** | **39.0** | **29.8** | **29.8** |
| **3. *D. mccartyi* CG4** | **48.70** | **98.54** | **97.93** |  | **81.9**  Values above the diagonal: dDDH | **84.2** | **38.8** | **29.7** | **29.6** |
| **4. *D.* *mccartyi* 195** | **48.90** | **97.84** | **97.71** | **97.90** |  | **79.4** | **38.5** | **30.2** | **30.2** |
| **5. *D. mccartyi* MB** | **48.30** | **97.90** | **97.83** | **98.10** | **97.72** |  | **39.1** | **29.4** | **29.4** |
| **6. *D. mccartyi* GY50** | **47.00** | **89.76** | **89.67** | **89.74** | **89.37** | **89.84** |  | **31.0** | **30.9** |
| **7. *D. mccartyi* KS** | **47.23** | **84.86** | **85.04** | **84.88** | **85.17** | **84.77** | **85.45**  Values below the diagonal: ANI |  | **99.20** |
| **8. *D. mccartyi* RC** | **47.17** | **84.75** | **84.99** | **84.80** | **85.24** | **84.77** | **85.45** | **99.87** |  |

The values greater than ANI and dDDH Thresholds (i.e., >95% and >70%) are considered to two strains belonging to be the same species. The Values above and below the diagonal indicate values of dDDH and ANI, respectively. Blue and red fonts represent respectively the values below ANI Threshold (i.e., 95%) and below dDDH Threshold (i.e., 70%).

**Table S5.** All Proteins expressed in 1,2-DCA-dechlorinating PJ_DCA_ culture and 1,2-DCP- or 1,1,2-TCA-dechlorinating PJ_TCA_ culture using the reference protein database of *Dhc* strains PJ_DCA_ and PJ_TCA_, respectively.

**(See Table S5.xlsx)**

**Table S6**. Summary of published halogenated substrates for *Dhc* spp.

| **Pure culture or cultures** | ***Dhc* spp.** | **Substrates for OHR → products** | **Group** | **References** |
| --- | --- | --- | --- | --- |
|  |  |  |  |  |
| **Pure culture** | MB | PCE, TCE → *t*DCE;  *Pe*BDEs → di- to tetra-BDEs;  PCBs ^a^ | Cornell | ^30, 31^ |
|  | ANAS1 | TCE, *c*DCE, 1,1-DCE → VC (ETH) |  | ^32^ |
|  | ANAS2 | TCE, *c*DCE, 1,1-DCE, VC → ETH |  | ^32^ |
|  | CG4 | PCBs (para > meta);  PCE → *t*DCE |  | ^33^ |
|  | GEO12 | 1,2-DCA → ETH;  TCE → ETH |  | ^34^ |
|  | 195 | PCE, TCE, *c*DCE, 1,1-DCE → VC (ETH) ^b^;  1,2-DCA → ETH;  2,3-DCP → 3-CP;  HCB → 1,2,4,5-*Te*CB, 1,3,5-TCB;  HCH →TeCCH → MCB, benzene |  | ^35-39^ |
|  | VS | TCE, *c*DCE, 1,1-DCE, VC → ETH | Victoria | ^15, 40^ |
|  | CG1 | PCBs (para > meta);  PCE → TCE;  β-TBCO →4,5-dibromo-9-oxabicyclo [6.1.0] nonane;  DPTE →2,3-dibromopropyl-2-bromophenyl ether;  *Pe*BDEs;  TBBPA |  | ^33, 41, 42^ |
|  | GY50 | TCE → *c*DCE;  2,4,6-TCP → 2,4-DCP;  *Pe*BDE, *Te*BDEs → diphenyl ether |  | ^43^ |
|  | BAV1 | DCEs, VC → ETH;  VBr → ETH | Pinellas | ^11, 44^ |
|  | FL2 | TCE, *c*DCE, *t*DCE → VC (ETH) ^b^ |  | ^45^ |
|  | GT | TCE, *c*DCE, 1,1-DCE → ETH |  | ^46^ |
|  | 11a | TCE, DCEs, VC → ETH;  1,2-DCA → ETH |  | ^13^ |
|  | 11a5 | TCE, *c*DCE, 1,1-DCE, *t*DCE → VC |  | ^13^ |
|  | TZ50 | PCE, TCE, DCEs → VC, ETH;  *Pe*BDE, *Te*BDEs → diphenyl ether |  | ^47^ |
|  | BTF08 | PCE → ETH;  1,2-DCA → ETH;  HCH →TeCCH → MCB, benzene |  | ^39, 48^ |
|  | CBDB1 | HCB → 1,4-DCB, 1,3-DCB, 1,3,5-TCB;  Dioxins; PCBs ^c^; chloroanilines;  *Pe*CP → TCPs + 2-CP + 3-CP;  Bromobenzenes → benzene;  PCE, TCE → *t*DCE;  D- and L-3,5-dibromotyrosine → tyrosine;  2,4,5-T → 2,4,5-TCP; |  | ^35, 49-55^ |
|  | DCMB | Dioxins;  1,2,3-TCB → 1,2-DCB |  | ^56^ |
|  | JNA | PCBs (flanked meta) |  | ^57^ |
|  | CG5 | PCBs (meta + para);  PCE → *c*DCE + *t*DCE |  | ^33^ |
| ***Dhc*-containing enrichment cultures** | RC & KS | 1,2-DCP → Propene |  | ^58^ |
|  | TCEP-transforming culture | TCEP → bis(2-chloroethyl) phosphate and ethene | NA | ^59, 60^ |
|  | TCPP-transforming culture | TCPP → bis(1-chloro-2-propyl) phosphate and propene |  | ^59^ |
|  | *Dhc* spp. | HCBD ^d^ → (*E*)-1,1,2,3,4-penta-CBD, (*Z, E*)-1,2,3,4-tetra-CBD, (*E*)-1,2,3-tri-CBD |  | ^61^ |
|  | *Dhc*- containing culture | 1,2-DBA → ETH |  | ^62^ |
|  | PB | *Pe*BDE → diphenyl ether |  | ^63^ |
|  | *Dhc*- containing culture | *Pe*BDEs;  TBBPA;  2,4,6-TCP |  | ^64^ |
|  | XH-1 | 4-CP → phenol |  | ^65^ |
|  | *Dhc*- containing culture | 6:2 Cl-PFESA → 6:2 H-PFESA ^e^ |  | ^66^ |
|  | *Dhc*- containing culture | PCDDs |  | ^67^ |
|  | *Dhc*- containing culture | α- and γ-HCH → TeCCH →benzene |  | ^68^ |
|  | strain 195- containing cultures | HBCD (α-, β-, and γ-) →TBCD, DBCD, and 1,5,9-cyclododecatriene | Cornell | ^69^ |

Abbreviations: *Pe* penta; *Te* tetra; A, ethane; B, benzene; P, phenol; VBr, vinyl bromide; BDE, brominated diphenyl ether; PCBs, polychlorinated biphenyls; MCB, monochlorobenzene; 2,4,6-TCP, 2,4,6-trichlorophenol; 2,4-DCP, 2,4-dichlorophenol; 1,2-DCP, dichlorophenol; HCH, hexachlorocyclohexane; TeCCH, tetrachlorocyclohexene; HCB, hexachlorobenzene; 1,4-DCB, 1,4-dichlorobenzene; 1,3-DCB, 1,3-dichlorobenzene; 1,2-DCB, dichlorobenzene; 1,3,5-TCB, 1,3,5-trichlorobenzene; 1,2,3-TCB, 1,2,3-trichlorobenzene; CP, chlorophenol; 2,4,5-T, 2,4,5-trichlorophenoxyacetic acid; 1,2-DCP, 1,2-dichloropropane; TCEP, tris(2-chloroethyl) phosphate; TCPP, tris(1-chloro-2-propyl) phosphate; HCBD, hexachloro-1,3-butadiene; 1,1,2,3,4-penta-CBD, 1,1,2,3,4-pentachloro-1,3-butadiene; 1,2,3,4-tetra-CBD, 1,2,3,4-tetrachloro-1,3-butadiene; 1,2,3-tri-CBD, 1,2,3-trichloro-1,3-butadiene; 1,2-DBA, 1,2-dibromoethane; TBCO, 1,2,5,6-tetrabromocyclooctane; DPTE, 2,3-dibromopropyl-2,4,6-tribromophenyl ether; TBBPA, tetrabromobisphenol A; 6:2 Cl-PFESA, 6:2 chlorinated polyfluorooctane ether sulfonate; 6:2 H-PFESA, 6:2 hydrogen-substituted polyfluorooctane ether sulfonate; PCDDs, dibenzo-p-dioxins; HBCD, hexabromocyclododecane; TBCD, tetrabromocyclododecene; DBCD, dibromocyclododecadiene;

^a^PCBs were dechlorinated in a co-metabolic way.

^b^VC → ethene cometabolic;

^c^Para and meta Cls removed from PCBs; growth using PCBs not demonstrated but likely.

^d^The dechlorination of HCBD to (E)-1,2,3-tri-CBD via (E)-1,1,2,3,4-penta-CBD and (Z, E)-1,2,3,4-tetra-CBD is in a co-metabolic way.

^e^6:2 Cl-PFESA → 6:2 H-PFESA possible cometabolic;

NA indicates the group information of *Dhc* strains in enriched cultures was not provided or analyzed in literature.

**Table S7**. Reported dihaloelimination RDase and associated substrate.

| **RDase** | **OHRB** | **Substrate** | | **Reference** |
| --- | --- | --- | --- | --- |
|  |  | **Dihaloelimination** | **Hydrogenolysis** |  |
| DcpA | *Dhgm* strain BL-DC-9 | 1,2-DCP, 1,2,3-TCP, 1,2-DCA, 1,1,2-TCA, 1,1,2,2-TeCA | ^ND^ | ^16, 17^ |
| DcpA | *Dhgm* strains IP3-3 and SPB-1 | 1,2-DCA, 1,1,2-TCA, 1,2-DCP, 1,2,3-TCP, 1,1,2,2-TeCA | ^ND^ | ^18^ |
| DcpA | *Dhc* strain RC | 1,2-DCP | ^NT^ | ^60^ |
| DcpA | *Dhc* strain KS | 1,2-DCP | ^NT^ | ^60^ |
| DcpA | *Dhgm* strain BRE15M | 1,2-DCP, 1,2,3-TCP, 1,2-DCA, 1,1,2-TCA, 1,1,2,2-TeCA, and 1,2-DBA | ^ND^ | ^19^ |
| DcpA | *Dhc* strains PJ_DCA_ and PJ_TCA_ | 1,2-DCP, 1,2,3-TCP, 1,2-DCA, 1,1,2-TCA, 1,1,2,2-TeCA, and 1,2-DBA | ^ND^ | This study |
| DcaA | *Desulfitobacterium* strain DCA1 | 1,2-DCA and all vicinal dichloropropanes and -butanes | PCE, TCE, TBE | ^21-23^ |
| DcaA | *Desulfitobacterium* strain AusDCA | 1,2-DCA | ^ND^ | ^23^ |
| DcaA | *Dhb* strain WL | 1,2-DCA and 1,1,2-TCA | PCE, TCE | ^24^ |
| TceA | *Dhc* strain 195 | 1,2-DCA | TCE, *c*DCE, 1,1-DCE | ^10^ |
| BvcA | *Dhc* strain BAV1 | 1,2-DCA | TCE, *c*DCE, *t*DCE, 1,1-DCE, VC | ^12, 70^ |
| VcrA | *Dhc* strain VS | 1,2-DCA | *c*DCE, *t*DCE, 1,1-DCE, VC | ^14, 15^ |
| VcrA | *Dhc* strain 11a | 1,2-DCA | TCE, *c*DCE, *t*DCE, 1,1-DCE, VC | ^13^ |
| DcrA | *Dhb* strain DCA | 1,1,2-TCA | 1,1-DCA | ^71^ |

Abbreviations: TBE, tribromoethene; 1,2-DBA, 1,2-dibromoethane.

^ND^ OHRB can’t degrade other substrates via hydrogenolysis.

^NT^ Other substrates were not tested.

**Table S8.** RDases with assigned functions and their associated OHRB.

| RDase | Host OHRB | Metabolic function(s) ^a^ | | GenBank accession number | How RDase function was determined? | Reference |
| --- | --- | --- | --- | --- | --- | --- |
|  |  | Substrate(s) | Product(s) |  |  |  |
| PceA | *Dhc* strain 195 | PCE | TCE | AAW40342 | Biochemical characterization | ^10^ |
| TceA | *Dhc* strain 195 | TCE, *c*DCE, 1,1-DCE, 1,2-DCA | VC (ethene) ^b^ | AAW39060 | Biochemical characterization | ^10^ |
| MbrA | *Dhc* strain MB | TCE | *t*DCE, *c*DCE | ADF96893 | Transcriptional analysis | ^30^ |
| BvcA | *Dhc* strain BAV1 | TCE, 1,2-DCA,  DCEs, VC | Ethene | AAT64888 | Transcriptional analysis, protein expression & Biochemical characterization | ^12, 70^ |
| VcrA | *Dhc* strain VS | DCEs, 1,2-DCA, VC | Ethene | WP_012882535 | Biochemical characterization | ^14, 15^ |
| DcpA | *Dhc* strain RC | 1,2-DCP | Propene | AGS15114 | Transcriptional analysis & protein expression | ^60^ |
| DcpA | *Dhc* strain KS | 1,2-DCP | Propene | AGS15112 | Transcriptional analysis & protein expression | ^60^ |
| PcbA | *Dhc* strain CG5 | *Meta*-, *para*-PCBs/ PCE | Less chlorinated PCBs/ TCE | AII60305 | Transcriptional analysis | ^33^ |
| CerA | ‘*Ca.* Dhgm etheniformans’ strain GP | *c*DCE, VC | Ethene | PMP99143 | Protein expression | ^72^ |
| TdrA | *Dhgm* sp. strain WBC-2 | *t*DCE | VC | AKG53095 | Protein expression | ^73^ |
| DcpA | *Dhgm lykanthroporepellens* strain BL-DC-9 | 1,2,3-TCP/1,2-DCP/ 1,2-DCA/  1,1,2-TCA | Allyl chloride/  Propene/ Ethene/  VC | WP_013218938 | Transcriptional analysis | ^17^ |
| DcpA | *Dhgm alkenigignens* strain BRE15M | 1,2,3-TCP/1,2-DCP/ 1,2-DCA/  1,1,2-TCA/1,2-DBA | Allyl chloride/  Propene/ Ethene/  VC/Ethene | WP_116633212 | Biochemical characterization | ^74^ |
| PceA | *Dhb restrictus* strain PER-K23 | PCE, TCE | *c*DCE | AHF10727 | Biochemical characterization | ^75^ |
| CfrA | *Dhb* sp. strain CF | CF/ 1,1,1-TCA | DCM/ 1,1-DCA | AFV05253 | Protein expression | ^70^ |
| PceA | *Sulfurospirillum multivorans* DSM 12446 | PCE, TCE | *c*DCE | AHJ12791 | Biochemical characterization | ^76^ |
| PceA | *Desulfitobacterium hafniense* strain Y51 | PCE, TCE | *c*DCE | BAE84628 | Biochemical characterization | ^77^ |
| PceA | *Desulfitobacterium* sp. strain PCE1 | PCE | TCE | AAG49543 | Biochemical characterization | ^78^ |
| CprA | *Desulfitobacterium hafniense* strain DCB-2 | Cl-HPA | HPA | AAG46192 | Biochemical characterization | ^79^ |
| DcaA | *Desulfitobacterium dichloroeliminans* strain DCA1 | 1,2-DCA | Ethene | **CAJ75430** | Biochemical characterization | ^23^ |
| CprA | *Desulfitobacterium chlororespirans* strain Co23 | *Ortho*-chlorophenols | Less chlorinated phenols | AAL84925 | Biochemical characterization | ^80^ |
| BhbA | *Comamonas* sp. strain 7D-2 | bromoxynil | 4-carboxy-2-hydroxymuconate-6-semialdehyde | AFV28965 | Biochemical characterization & molecular characterization | ^81^ |
| CprA | *Desulfitobacterium* sp. Viet-1 | 2,4-DCP | 4-CP | AAG49544 | Phylogenetic inference | ^82^ |
| CrdA | *Desulfitobacterium hafniense* strain PCP-1 | 2,4,6-TCP | 2,4-DCP | AAK95329 | Biochemical characterization & transcriptional analysis | ^83, 84^ |
| NpRdhA | *Nitratireductor pacificus* strain pht-3B | 35-DB-4-OH | 3-B-4-OH | EKF18105 | Enzyme assays, heterologous expression, molecular characterization | ^85^ |
| PceA | *Sulfurospirillum multivorans* strain N | PCE, TCE | *c*DCE | AAC60788 | Transcriptional analysis | ^86^ |
| PceA-DCE | *Sulfurospirillum* mixed culture SL2 | PCE, TCE | *c*DCE | AGW23613 | Enzyme assays | ^87^ |
| PceA-TCE | *Sulfurospirillum* mixed culture SL2 | PCE | *c*DCE | AGW23615 | Enzyme assays | ^87^ |
| TcbA | *Dhb* sp. strain TeCB1 | 1,2,3,4-TeCB,  1,2,4-TCB | 1,3-DCB,  1,4-DCB | WP_068882928 | Biochemical characterization | ^88^ |
| TmrA | *Dhb* sp. strain UNSWDHB | CF/ 1,1,2-TCA/ 1,1,1-TCA/ 1,1-DCA | DCM/ 1,2-DCA and VC/ 1,1-DCA/ CA | WP_034377773 | Biochemical characterization & Enzyme kinetic experiments | ^29^ |
| VcrA | *Dhc* strain WBC-2 | VC | Ethene | AOV99943 | Phylogenetic inference | ^89^ |

OHRB abbreviations: *Dhc*, *Dehalococcoides mccartyi*; *Dhgm*, *Dehalogenimonas*; *Dhb*, *Dehalobacter*.

Organohalogen compound abbreviations: 1,2-DCA, 1,2-dichloroethane; 1,2-DCP, 1,2-dichloropropane; 1,1,2-TCA; 1,1,2,-trichloroethane; 1,1-DCA, 1,1-dichloroethane; CA, chloroethane; 1,2,3,4-TeCB, 1,2,3,4-tetrachlorobenzene; 1,2,3-TCB, 1,2,3-trichlorobenzene; 1,2,4-TCB, 1,2,4-trichlorobenzene; 1,3-DCB, 1,3-dichlorobenzene; 1,4-DCB, 1,4-dichlorobenzene; CF, chloroform; DCM, dichloromethane; 1,1,1-TCA, 1,1,1-trichloroethane; Cl-HPA, chloro-hydroxyphenylacetate; HPA, hydroxyphenylacetate; 1,2,3-TCP, 1,2,3-trichloropropane; 1,2-DCP, 1,2-dichloropropane; 2,4,6-TCP, 2,4,6-trichlorophenol; TCPs, trichlorophenols; 2,4-DCP, 2,4-dichlrophenol; 2-CP, 2-chlorophenol; 3-CP, 3-chlorophenol; PCB, polychlorinated biphenyl; MCB, monochlorobenzene; 35-DB-4-OH, 3,5-dibromo-4-hydroxybenzoic acid; 3-B-4-OH, 3-bromo-4-hydroxybenzoic acid.

^a^ RDase metabolic function is inferred based on expression studies in cultures that dechlorinated different chlorinated ethenes as electron acceptors and/or data generated in cell-free enzyme assays.

^b^ Ethene was produced as a co-metabolic product during TCE dechlorination.

**Table S9.** Nucleotide sequences of the primers and probes used in this study.

| **Primer or probe** | **Sequence (5’-3’)** | **Purpose** | **Target gene** | **Amplicon size (bp)** | **Annealing temperature** | **Reference** |
| --- | --- | --- | --- | --- | --- | --- |
| V3-V4-F | CCTACGGRRBGCASCAGKVRVGAAT ^a^ | PCR | Bacterial and archaeal 16S rRNA | 350 | 57 ℃ | ^90^ |
| V3-V4-R | GGACTACNVGGGTWTCTAATCC ^a^ |  |  |  |  |  |
| Dhc-730F | GCGGTTTTCTAGGTTGTC | PCR | *Dhc* 16S rRNA | 621 | 55 ℃ | ^44^ |
| Dhc-1350R | CACCTTGCTGATATGCGG |  |  |  |  |  |
| BL-DC-142F | GTGGGGGATAACACTTCGAAAGAAGTGC | PCR | *Dhgm* 16S rRNA | 1120 | 60 ℃ | ^91^ |
| BL-DC-1351R | AACGCGCTATGCTGACACGCGT |  |  |  |  |  |
| dcpA-360F | TTGCGTGATCAAATTGGAGCCTGG | PCR | *dcpA* | 1090 | 58 ℃ | ^60^ |
| dcpA-1449R | TTTAAACAGCGGGCAGGTACTGGT |  |  |  |  |  |
| Dhc1200F | CTGGAGCTAATCCCCAAAGCT | qPCR | *Dhc* 16S rRNA | 66 | 60 ℃ | ^92^ |
| Dhc1271R | CAACTTCATGCAGGCGGG |  |  |  |  |  |
| Dhc1240Probe | 6FAM-TCGGATTGCAGGCTGA-MGB ^b^ |  |  |  |  |  |

^a^ Degenerate bases, R=A/G, B=C/G/T, S=C/G, K=G/T, V=A/C/G, N=A/C/G/T, W=A/T.

^b^ 6FAM, 6-carboxyfluorescein; MGB, minor groove binder moiety.


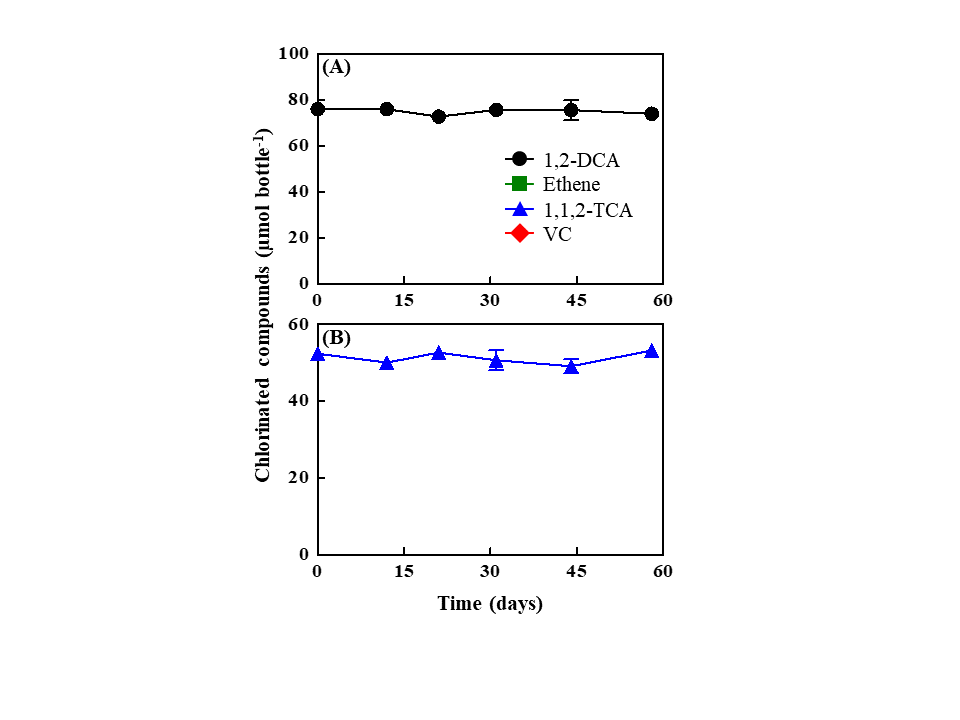


**Figure S1.** No dechlorination and degradation of 1,2-DCA (A) and 1,1,2-TCA (B) in heat-killed negative control incubations. Error bars represent standard deviation of triplicate bottles.


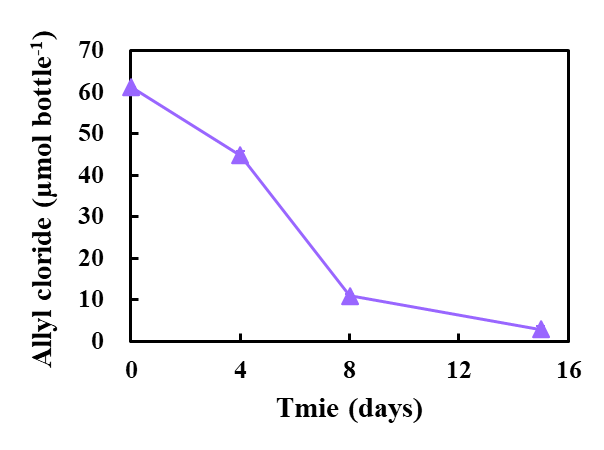


**Figure S2.** Abiotic transformation of allyl chloride in the medium. Error bars represent standard deviation of triplicate bottles.


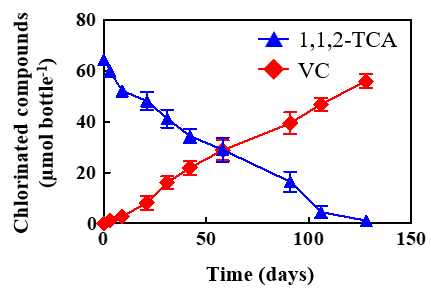


Figure S3. Reductive dechlorination of 1,1,2-TCA in the 3th generation PJ_TCA_ culture amended with acetate. The error bars represent standard deviations of triplicate bottles and are not displayed when smaller than the symbol.


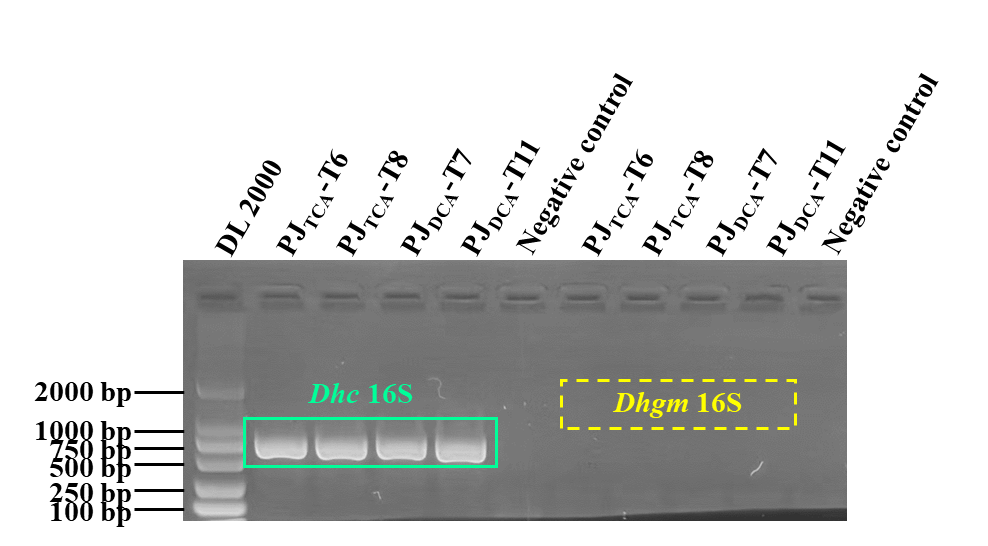


**Figure S4.** Gel electrophoresis of PCR amplicons generated using template DNA extracted from the PJ_DCA_ or PJ_TCA_ culture.


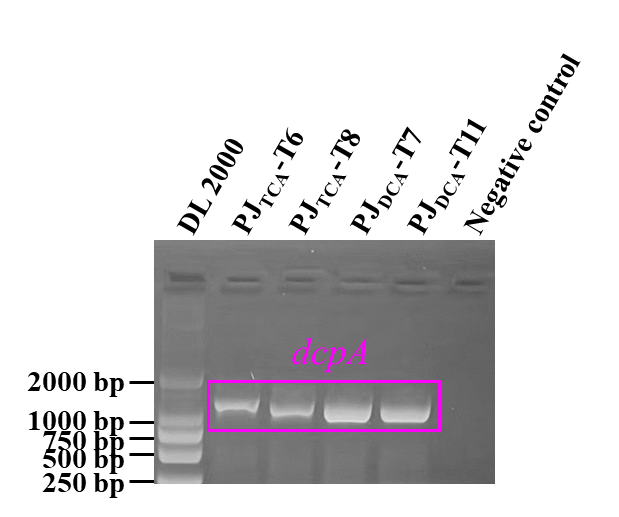


**Figure S5.** Gel electrophoresis of PCR amplicons generated using template DNA extracted from the PJ_DCA_ or PJ_TCA_ culture.

**
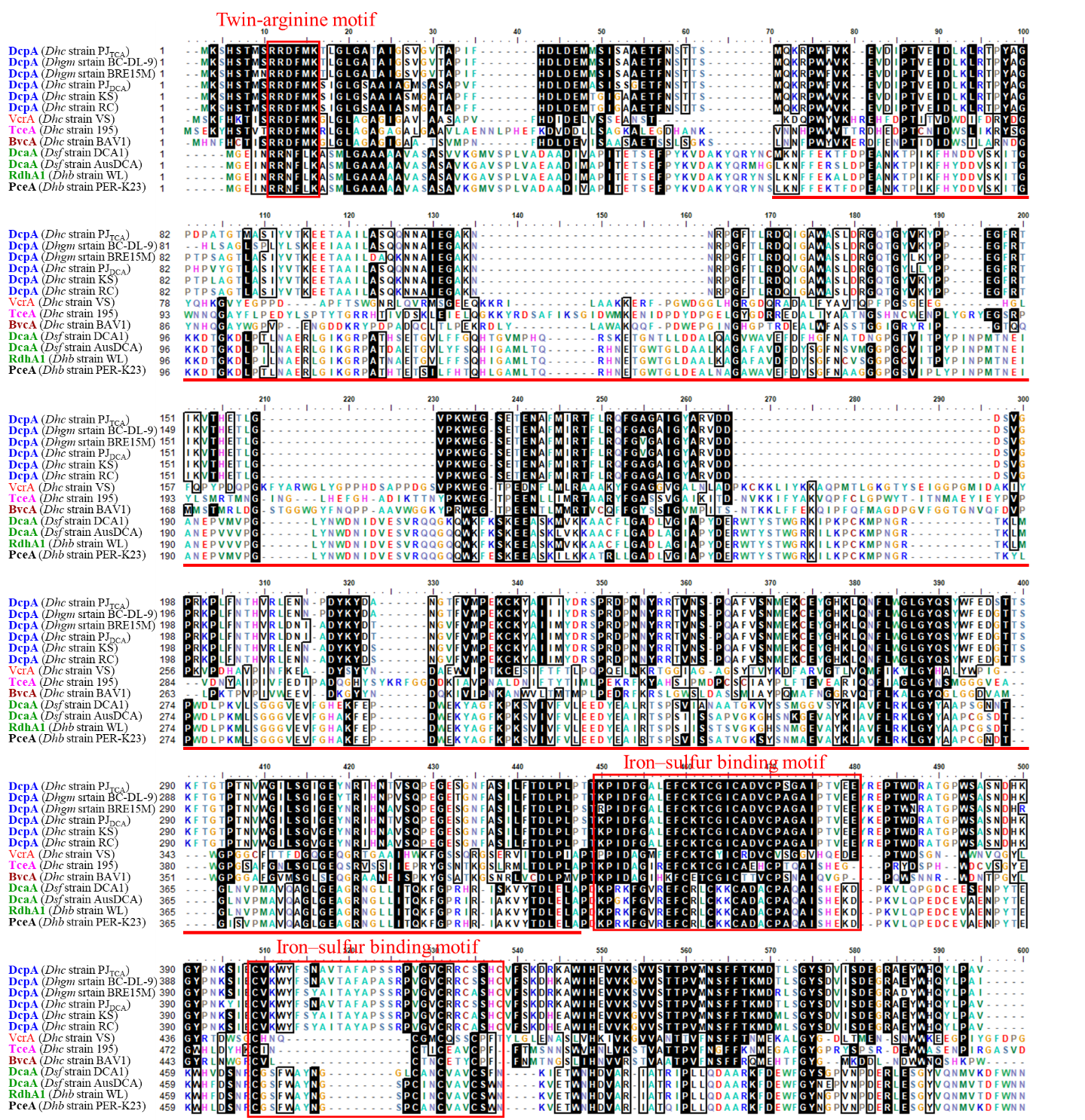
**

**Figure S6.** Multiple sequence alignment of characterized 1,2-DCA-/1,1,2-TCA- and/or 1,2-DCP-dihaloeliminating RDases, including 6 DcpA-like RDases, 3 DcaA-like RDases, VcrA, TceA, and BvcA. PceA from *Dhb* strain PER-K23 was chose as outgroup that can’t dechlorinate 1,2-DCA and 1,1,2-TCA. Three conserved domains of RDase are marked in red boxes, which are twin-arginine motif (i.e., RRDFMK) near the N terminus and two iron–sulfur cluster binding motifs (i.e., FCX_2_CX_2_CX_3_CP and CX_2_CX_3_C) near the C terminus.

**
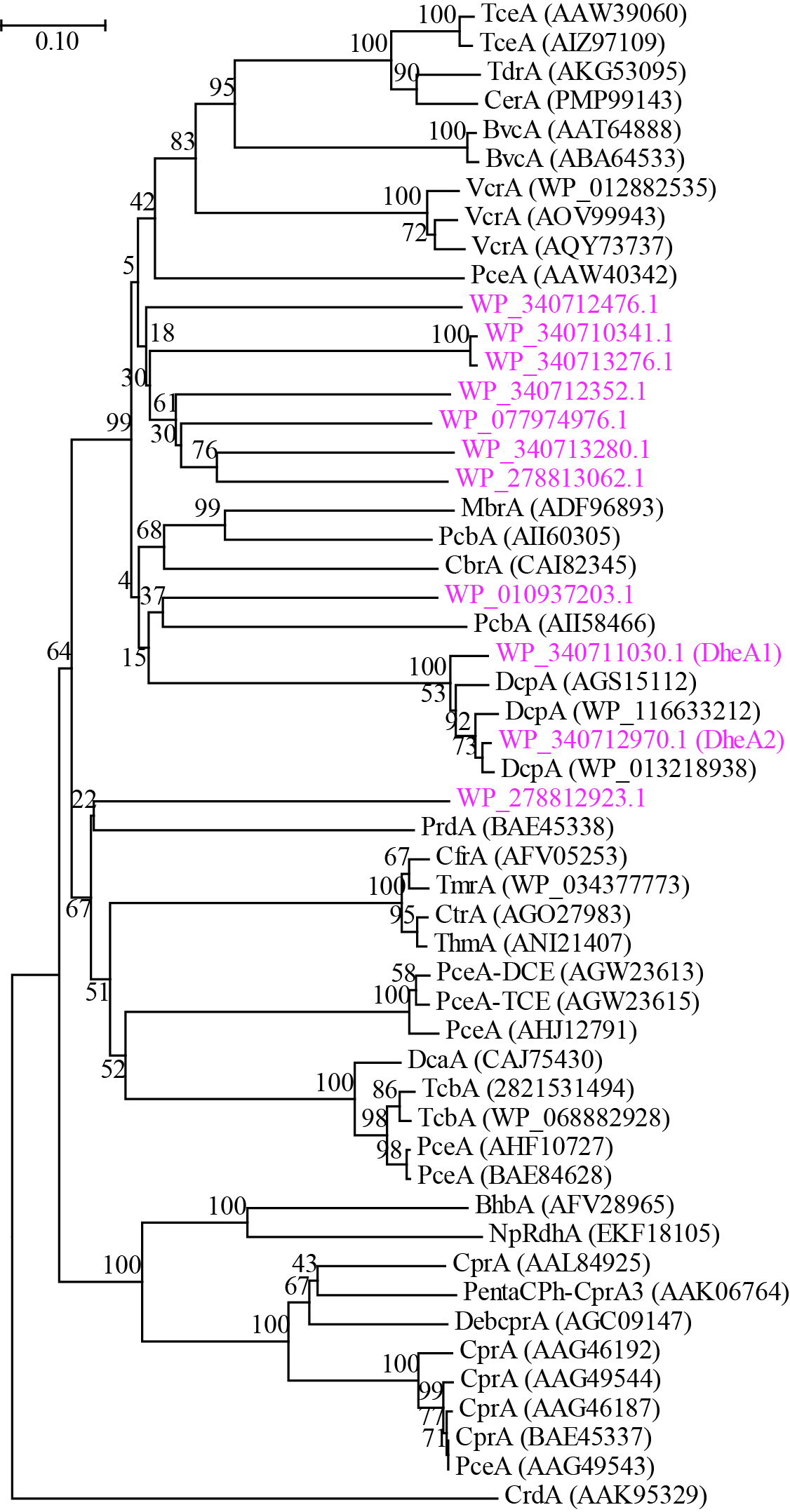
**

**Figure S7.** Phylogenetic relationship of 11 RDases expressed in strains PJ_DCA_ and PJ_TCA_ with 41 characterized RDases. The pink fonts represent the RDases expressed in strains PJ_DCA_ and PJ_TCA_ grown with 1,2-DCA, 1,1,2-TCA, or 1,2-DCP. Numbers at the branch points represent bootstrap percentages calculated from 1,000 replicate trees. The scale bar represents the amino acid sequence divergence. GenBank accession numbers are provided in parentheses.

**Reference**

1. Kozich, J. J.; Westcott, S. L.; Baxter, N. T.; Highlander, S. K.; Schloss, P. D. Development of a dual-index sequencing strategy and curation pipeline for analyzing amplicon sequence data on the MiSeq Illumina sequencing platform. *Appl. Environ. Microbiol.* **2013,** *79*, 5112-5120.

2. Edgar, R. C.; Haas, B. J.; Clemente, J. C.; Quince, C.; Knight, R. UCHIME improves sensitivity and speed of chimera detection. *Bioinformatics* **2011,** *27*, 2194-2200.

3. Haas, B. J.; Gevers, D.; Earl, A. M.; Feldgarden, M.; Ward, D. V.; Giannoukos, G.; Ciulla, D.; Tabbaa, D.; Highlander, S. K.; Sodergren, E.; Methé, B.; DeSantis, T. Z.; Petrosino, J. F.; Knight, R.; Birren, B. W. Chimeric 16S rRNA sequence formation and detection in Sanger and 454-pyrosequenced PCR amplicons. *Genome Res.* **2011,** *21*, 494-504.

4. Martin, M. Cutadapt removes adapter sequences from high-throughput sequencing reads. *EMBnet.journal* **2011,** *17*, 10-12.

5. Jiang, L.; Yang, Y.; Jin, H.; Wang, H.; Swift, C. M.; Xie, Y.; Schubert, T.; Loffler, F. E.; Yan, J. *Geobacter* sp. strain IAE dihaloeliminates 1,1,2-trichloroethane and 1,2-dichloroethane. *Environ. Sci. Technol.* **2022,** *56*, 3430-3440.

6. Walker, S. E.; Lorsch, J. Sanger dideoxy sequencing of DNA. *Methods Enzymol.* **2013,** *529*, 171-184.

7. Löffler, F. E.; Yan, J.; Ritalahti, K. M.; Adrian, L.; Edwards, E. A.; Konstantinidis, K. T.; Muller, J. A.; Fullerton, H.; Zinder, S. H.; Spormann, A. M. *Dehalococcoides mccartyi* gen. nov., sp. nov., obligately organohalide-respiring anaerobic bacteria relevant to halogen cycling and bioremediation, belong to a novel bacterial class, *Dehalococcoidia* classis nov., order *Dehalococcoidales* ord. nov. and family *Dehalococcoidaceae* fam. nov., within the phylum *Chloroflexi*. *Int. J. Syst. Evol. Microbiol.* **2013,** *63*, 625-635.

8. Kumar, S.; Stecher, G.; Tamura, K. MEGA7: molecular evolutionary genetics analysis version 7.0 for bigger datasets. *Mol. Biol. Evol.* **2016,** *33*, 1870-1874.

9. Maymó-Gatell, X.; Anguish, T.; Zinder, S. H. Reductive dechlorination of chlorinated ethenes and 1, 2-dichloroethane by "*Dehalococcoides ethenogenes*" 195. *Appl. Environ. Microbiol.* **1999,** *65*, 3108-3113.

10. Magnuson, J. K.; Romine, M. F.; Burris, D. R.; Kingsley, M. T. Trichloroethene reductive dehalogenase from *Dehalococcoides ethenogenes*: sequence of *tceA* and substrate range characterization. *Appl. Environ. Microbiol.* **2000,** *66*, 5141-5147.

11. He, J.; Ritalahti, K. M.; Yang, K. L.; Koenigsberg, S. S.; Löffler, F. E. Detoxification of vinyl chloride to ethene coupled to growth of an anaerobic bacterium. *Nature* **2003,** *424*, 62-65.

12. Krajmalnik-Brown, R.; Hölscher, T.; Thomson, I. N.; Saunders, F. M.; Ritalahti, K. M.; Löffler, F. E. Genetic identification of a putative vinyl chloride reductase in *Dehalococcoides* sp. strain BAV1. *Appl. Environ. Microbiol.* **2004,** *70*, 6347-6351.

13. Lee, P. K.; Cheng, D.; West, K. A.; Alvarez-Cohen, L.; He, J. Isolation of two new *Dehalococcoides mccartyi* strains with dissimilar dechlorination functions and their characterization by comparative genomics via microarray analysis. *Environ. Microbiol.* **2013,** *15*, 2293-2305.

14. Parthasarathy, A.; Stich, T. A.; Lohner, S. T.; Lesnefsky, A.; Britt, R. D.; Spormann, A. M. Biochemical and EPR-spectroscopic investigation into heterologously expressed vinyl chloride reductive dehalogenase (VcrA) from *Dehalococcoides mccartyi* strain VS. *J. Am. Chem. Soc.* **2015,** *137*, 3525-3532.

15. Müller, J. A.; Rosner, B. M.; Von Abendroth, G.; Meshulam-Simon, G.; McCarty, P. L.; Spormann, A. M. Molecular identification of the catabolic vinyl chloride reductase from *Dehalococcoides* sp. strain VS and its environmental distribution. *Appl. Environ. Microbiol.* **2004,** *70*, 4880-4888.

16. Yan, J.; Rash, B. A.; Rainey, F. A.; Moe, W. M. Isolation of novel bacteria within the *Chloroflexi* capable of reductive dechlorination of 1,2,3-trichloropropane. *Method Enzymol.* **2009,** *11*, 833-843.

17. Siddaramappa, S.; Challacombe, J. F.; Delano, S. F.; Green, L. D.; Daligault, H.; Bruce, D.; Detter, C.; Tapia, R.; Han, S.; Goodwin, L.; Han, J.; Woyke, T.; Pitluck, S.; Pennacchio, L.; Nolan, M.; Land, M.; Chang, Y.-J.; Kyrpides, N. C.; Ovchinnikova, G.; Hauser, L.; Lapidus, A.; Yan, J.; Bowman, K. S.; da Costa, M. S.; Rainey, F. A.; Moe, W. M. Complete genome sequence of *Dehalogenimonas lykanthroporepellens* type strain (BL-DC-9^T^) and comparison to "*Dehalococcoides*" strains. *Stand. Genomic Sci.* **2012,** *6*, 251-264.

18. Bowman, K. S.; Nobre, M. F.; da Costa, M. S.; Rainey, F. A.; Moe, W. M. *Dehalogenimonas alkenigignens* sp. nov., a chlorinated-alkane-dehalogenating bacterium isolated from groundwater. *Int. J. Syst. Evol. Microbiol.* **2013,** *63*, 1492-1498.

19. Martín-González, L.; Mortan, S. H.; Rosell, M.; Parladé, E.; Martínez-Alonso, M.; Gaju, N.; Caminal, G.; Adrian, L.; Marco-Urrea, E. Stable carbon isotope fractionation during 1,2-dichloropropane-to-propene transformation by an enrichment culture containing *Dehalogenimonas* strains and a *dcpA* gene. *Environ. Sci. Technol.* **2015,** *49*, 8666-8674.

20. Cui, Y.; Li, X.; Yan, J.; Lv, Y.; Jin, H.; Wang, J.; Chen, G.; Kara-Murdoch, F.; Yang, Y.; Löffler, F. E. *Dehalogenimonas etheniformans* sp. nov., a formate-oxidizing, organohalide-respiring bacterium isolated from grape pomace. *Int. J. Syst. Evol. Microbiol.* **2023,** *73*, 005881.

21. Marzorati, M.; de Ferra, F.; Van Raemdonck, H.; Borin, S.; Allifranchini, E.; Carpani, G.; Serbolisca, L.; Verstraete, W.; Boon, N.; Daffonchio, D. A novel reductive dehalogenase, identified in a contaminated groundwater enrichment culture and in *Desulfitobacterium dichloroeliminans* strain DCA1, is linked to dehalogenation of 1,2-dichloroethane. *Appl. Environ. Microbiol.* **2007,** *73*, 2990-2999.

22. De Wildeman, S.; Diekert, G.; Van Langenhove, H.; Verstraete, W. Stereoselective microbial dehalorespiration with vicinal dichlorinated alkanes. *Appl. Environ. Microbiol.* **2003,** *69*, 5643-5647.

23. Low, A.; Zhao, S.; Rogers, M. J.; Zemb, O.; Lee, M.; He, J.; Manefield, M. Isolation, characterization and bioaugmentation of an acidotolerant 1,2-dichloroethane respiring *Desulfitobacterium* species from a low pH aquifer. *FEMS Microbiol. Ecol.* **2019,** *95*, fiz055.

24. Grostern, A.; Edwards, E. A. Growth of *Dehalobacter* and *Dehalococcoides* spp. during degradation of chlorinated ethanes. *Appl. Environ. Microb.* **2006,** *72*, 428-436.

25. Yoshida, N. G., A. Geobacter bacteria that detoxify 1,2-dichloroethane to ethylene. JP 5764880 B2, **2015**.

26. Zhao, S.; Ding, C.; He, J., Detoxification of 1,1,2-trichloroethane to ethene by *Desulfitobacterium* and identification of its functional reductase gene. *PLoS One* **2015,** *10*, e0119507.

27. Wang, P. H.; Tang, S.; Nemr, K.; Flick, R.; Yan, J.; Mahadevan, R.; Yakunin, A. F.; Löffler, F. E.; Edwards, E. A. Refined experimental annotation reveals conserved corrinoid autotrophy in chloroform-respiring *Dehalobacter* isolates. *Isme J.* **2017,** *11*, 626-640.

28. Soder-Walz, J. M.; Torrentó, C.; Algora, C.; Wasmund, K.; Cortés, P.; Soler, A.; Vicent, T.; Rosell, M.; Marco-Urrea, E. Trichloromethane dechlorination by a novel *Dehalobacter* sp. strain 8M reveals a third contrasting C and Cl isotope fractionation pattern within this genus. *Sci. Total Environ.* **2022,** *813*, 152659.

29. Wong, Y. K.; Holland, S. I.; Ertan, H.; Manefield, M.; Lee, M. Isolation and characterization of *Dehalobacter* sp. strain UNSWDHB capable of chloroform and chlorinated ethane respiration. *Environ. Microbiol.* **2016,** *18*, 3092-3105.

30. Cheng, D.; He, J. Isolation and characterization of "*Dehalococcoides*" sp. strain MB, which dechlorinates tetrachloroethene to trans-1,2-dichloroethene. *Appl. Environ. Microbiol.* **2009,** *75*, 5910-5918.

31. Xu, G.; Zhao, S.; Chen, C.; Zhao, X.; Ramaswamy, R.; He, J. Dehalogenation of polybrominated diphenyl ethers and polychlorinated biphenyls catalyzed by a reductive dehalogenase in *Dehalococcoides mccartyi* strain MB. *Environ. Sci. Technol.* **2022,** *56*, 4039-4049.

32. Lee, P. K.; Cheng, D.; Hu, P.; West, K. A.; Dick, G. J.; Brodie, E. L.; Andersen, G. L.; Zinder, S. H.; He, J.; Alvarez-Cohen, L. Comparative genomics of two newly isolated *Dehalococcoides* strains and an enrichment using a genus microarray. *Isme J.* **2011,** *5*, 1014-1024.

33. Wang, S.; Zhang, W.; Yang, K. L.; He, J. Isolation and characterization of a novel *Dehalobacter* species strain TCP1 that reductively dechlorinates 2,4,6-trichlorophenol. *Biodegradation* **2014,** *25*, 313-323.

34. Ding, C.; Rogers, M. J.; He, J. *Dehalococcoides mccartyi* strain GEO12 has a natural tolerance to chloroform inhibition. *Environ. Sci. Technol.* **2020,** *54*, 8750-8759.

35. Adrian, L.; Hansen, S. K.; Fung, J. M.; Gorisch, H.; Zinder, S. H., Growth of *Dehalococcoides* strains with chlorophenols as electron acceptors. *Environ. Sci. Technol.* **2007,** *41*, 2318-2323.

36. Fennell, D. E.; Nijenhuis, I.; Wilson, S. F.; Zinder, S. H.; Häggblom, M. M. *Dehalococcoides ethenogenes* strain 195 reductively dechlorinates diverse chlorinated aromatic pollutants. *Environ. Sci. Technol.* **2004,** *38*, 2075-2081.

37. Maymó-Gatell, X.; Chien, Y.; Gossett, J. M.; Zinder, S. H. Isolation of a bacterium that reductively dechlorinates tetrachloroethene to ethene. *Science* **1997,** *276*, 1568-71.

38. Maymó-Gatell, X.; Nijenhuis, I.; Zinder, S. H. Reductive dechlorination of cis-1,2-dichloroethene and vinyl chloride by "*Dehalococcoides ethenogenes*". *Environ. Sci. Technol.* **2001,** *35*, 516-521.

39. Bashir, S.; Kuntze, K.; Vogt, C.; Nijenhuis, I. Anaerobic biotransformation of hexachlorocyclohexane isomers by *Dehalococcoides* species and an enrichment culture. *Biodegradation* **2018,** *29*, 409-418.

40. Cupples, A. M.; Spormann, A. M.; McCarty, P. L. Growth of a *Dehalococcoides*-like microorganism on vinyl chloride and cis-dichloroethene as electron acceptors as determined by competitive PCR. *Appl. Environ. Microbiol.* **2003,** *69*, 953-959.

41. Huang, C.; Zeng, Y.; Hu, K.; Jiang, Y.; Zhang, Y.; Lu, Q.; Liu, Y. E.; Gao, S.; Wang, S.; Luo, X.; Mai, B. Anaerobic biotransformation of two novel brominated flame retardants: Kinetics, isotope fractionation and reaction mechanisms. *Water Res.* **2023,** *243*, 120360.

42. Ramaswamy, R.; Zhao, S.; Bae, S.; He, J. Debromination of TetraBromoBisphenol-A (TBBPA) depicting the metabolic versatility of *Dehalococcoides*. *J. Hazard. Mater.* **2021,** *419*, 126408.

43. Ding, C.; Rogers, M. J.; Yang, K. L.; He, J. Loss of the ssrA genome island led to partial debromination in the PBDE respiring *Dehalococcoides mccartyi* strain GY50. *Environ. Microbiol.* **2017,** *19*, 2906-2915.

44. He, J.; Ritalahti, K. M.; Aiello, M. R.; Löffler, F. E. Complete detoxification of vinyl chloride by an anaerobic enrichment culture and identification of the reductively dechlorinating population as a *Dehalococcoides* species. *Appl. Environ. Microbiol.* **2003,** *69*, 996-1003.

45. He, J.; Sung, Y.; Krajmalnik-Brown, R.; Ritalahti, K. M.; Löffler, F. E. Isolation and characterization of *Dehalococcoides* sp. strain FL2, a trichloroethene (TCE)- and 1,2-dichloroethene-respiring anaerobe. *Environ. Microbiol.* **2005,** *7*, 1442-1450.

46. Sung, Y.; Ritalahti, K. M.; Apkarian, R. P.; Löffler, F. E. Quantitative PCR confirms purity of strain GT, a novel trichloroethene-to-ethene-respiring *Dehalococcoides* isolate. *Appl. Environ. Microbiol.* **2006,** *72*, 1980-1987.

47. Zhao, S.; Rogers, M. J.; Cao, L.; Ding, C.; He, J. Identification of reductive dehalogenases that mediate complete debromination of penta- and tetrabrominated diphenyl ethers in *Dehalococcoides* spp. *Appl. Environ. Microbiol.* **2021,** *87*, e0060221.

48. Franke, S.; Seidel, K.; Adrian, L.; Nijenhuis, I. Dual element (C/Cl) isotope analysis indicates distinct mechanisms of reductive dehalogenation of chlorinated ethenes and dichloroethane in *Dehalococcoides mccartyi* strain BTF08 with defined reductive dehalogenase inventories. *Front. Microbiol.* **2020,** *11*, 1507.

49. Adrian, L.; Szewzyk, U.; Wecke, J.; Gorisch, H. Bacterial dehalorespiration with chlorinated benzenes. *Nature* **2000,** *408*, 580-583.

50. Adrian, L.; Dudková, V.; Demnerová, K.; Bedard, D. L. "*Dehalococcoides*" sp. strain CBDB1 extensively dechlorinates the commercial polychlorinated biphenyl mixture aroclor 1260. *Appl. Environ. Microbiol.* **2009,** *75*, 4516-4524.

51. Bunge, M.; Adrian, L.; Kraus, A.; Opel, M.; Lorenz, W. G.; Andreesen, J. R.; Görisch, H.; Lechner, U. Reductive dehalogenation of chlorinated dioxins by an anaerobic bacterium. *Nature* **2003,** *421*, 357-360.

52. Jayachandran, G.; Gorisch, H.; Adrian, L. Dehalorespiration with hexachlorobenzene and pentachlorobenzene by *Dehalococcoides* sp. strain CBDB1. *Arch. Microbiol.* **2003,** *180*, 411-416.

53. Wagner, A.; Cooper, M.; Ferdi, S.; Seifert, J.; Adrian, L. Growth of *Dehalococcoides mccartyi* strain CBDB1 by reductive dehalogenation of brominated benzenes to benzene. *Environ. Sci. Technol.* **2012,** *46*, 8960-8968.

54. Reino, C.; Ding, C.; Adrian, L. Continuous cultivation of *Dehalococcoides mccartyi* with brominated tyrosine avoids toxic byproducts and gives tight reactor control. *Water Res.* **2023,** *229*, 119396.

55. Zhang, S.; Wondrousch, D.; Cooper, M.; Zinder, S. H.; Schüürmann, G.; Adrian, L. Anaerobic dehalogenation of chloroanilines by *Dehalococcoides mccartyi* strain CBDB1 and *Dehalobacter* strain 14DCB1 via different pathways as related to molecular electronic structure. *Environ. Sci. Technol.* **2017,** *51*, 3714-3724.

56. Pöritz, M.; Goris, T.; Wubet, T.; Tarkka, M. T.; Buscot, F.; Nijenhuis, I.; Lechner, U.; Adrian, L. Genome sequences of two dehalogenation specialists – *Dehalococcoides mccartyi* strains BTF08 and DCMB5 enriched from the highly polluted Bitterfeld region. *FEMS Microbiol. Lett.* **2013,** *343*, 101-104.

57. LaRoe, S. L.; Fricker, A. D.; Bedard, D. L. *Dehalococcoides mccartyi* strain JNA in pure culture extensively dechlorinates Aroclor 1260 according to polychlorinated biphenyl (PCB) dechlorination Process N. *Environ. Sci. Technol.* **2014,** *48*, 9187-9196.

58. Loffler, F. E.; Champine, J. E.; Ritalahti, K. M.; Sprague, S. J.; Tiedje, J. M. Complete reductive dechlorination of 1,2-dichloropropane by anaerobic bacteria. *Appl. Environ. Microbiol.* **1997,** *63*, 2870-2875.

59. Zhu, X.; Deng, S.; Fang, Y.; Yang, S.; Zhong, Y.; Li, D.; Wang, H.; Wu, J.; Peng, P. *Dehalococcoides*-containing enrichment cultures transform two chlorinated organophosphate esters. *Environ. Sci. Technol.* **2022,** *56*, 1951-1962.

60. Padilla-Crespo, E.; Yan, J.; Swift, C.; Wagner, D. D.; Chourey, K.; Hettich, R. L.; Ritalahti, K. M.; Löffler, F. E. Identification and environmental distribution of dcpA, which encodes the reductive dehalogenase catalyzing the dichloroelimination of 1,2-dichloropropane to propene in organohalide-respiring *Chloroflexi*. *Appl. Environ. Microbiol.* **2014,** *80*, 808-818.

61. Shen, R.; Zhang, S.; Liang, Z.; Mai, B.; Wang, S. Mechanistic insight into co-metabolic dechlorination of hexachloro-1,3-butadiene in *Dehalococcoides*. *Water Res.* **2022,** *220*, 118725.

62. Palau, J.; Trueba-Santiso, A.; Yu, R.; Mortan, S. H.; Shouakar-Stash, O.; Freedman, D. L.; Wasmund, K.; Hunkeler, D.; Marco-Urrea, E.; Rosell, M. Dual C-Br isotope fractionation indicates distinct reductive dehalogenation mechanisms of 1,2-dibromoethane in *Dehalococcoides*- and *Dehalogenimonas*-containing cultures. *Environ. Sci. Technol.* **2023,** *57*, 1949-1958.

63. Zhao, S.; Fan, S.; He, Y.; Zhang, Y. Microbial debromination of polybrominated diphenyl ethers by *Dehalococcoides*-containing enrichment culture. *Front. Microbiol.* **2021,** *12*, 806795.

64. Xu, G.; Zhang, N.; Zhao, X.; Chen, C.; Zhang, C.; He, J. Offshore marine sediment microbiota respire structurally distinct organohalide pollutants. *Environ. Sci. Technol.* **2022,** *56*, 3065-3075.

65. Sun, B. H.; Song, X. Y.; Yan, J.; Yang, Y.; Lyu, Y.; Wang, J. J.; Song, Y. F.; Li, X. Y. [Degradation characteristics of 2,4,6-trichlorophenol by the anaerobic consortium XH-1]. *Ying Yong Sheng Tai Xue Bao* **2022,** *33*, 3395-3402.

66. Yi, S.; Morson, N.; Edwards, E. A.; Yang, D.; Liu, R.; Zhu, L.; Mabury, S. A. Anaerobic microbial dechlorination of 6:2 chlorinated polyfluorooctane ether sulfonate and the underlying mechanisms. *Environ. Sci. Technol.* **2022,** *56*, 907-916.

67. Zhang, S.; Li, Y.; Wang, S. Microbial reductive dechlorination of polychlorinated dibenzo-p-dioxins: Pathways and features unravelled via electron density. *J. Hazard. Mater.* **2022,** *424*, 127673.

68. Liu, Y.; Liu, J.; Renpenning, J.; Nijenhuis, I.; Richnow, H. H. Dual C-Cl isotope analysis for characterizing the reductive dechlorination of α- and γ-hexachlorocyclohexane by two *Dehalococcoides mccartyi* strains and an enrichment culture. *Environ. Sci. Technol.* **2020,** *54*, 7250-7260.

69. Zhong, Y.; Wang, H.; Yu, Z.; Geng, X.; Chen, C.; Li, D.; Zhu, X.; Zhen, H.; Huang, W.; Fennell, D. E.; Young, L. Y.; Peng, P. Diastereoisomer-specific biotransformation of hexabromocyclododecanes by a mixed culture containing *Dehalococcoides mccartyi* strain 195. *Front. Microbiol.* **2018,** *9*, 1713.

70. Tang, S.; Edwards, E. A. Identification of *Dehalobacter* reductive dehalogenases that catalyse dechlorination of chloroform, 1,1,1-trichloroethane and 1,1-dichloroethane. *Phil. Trans. R. Soc. B* **2013,** *368*, 20120318.

71. Picott, K. J.; Bowers, C. M.; Edwards, E. A. Deciphering reductive dehalogenase specificity through targeted mutagenesis of chloroalkane reductases. *Appl Environ Microbiol* **2025,** *91*, e0150124.

72. Yang, Y.; Higgins, S. A.; Yan, J.; Şimşir, B.; Chourey, K.; Iyer, R.; Hettich, R. L.; Baldwin, B.; Ogles, D. M.; Löffler, F. E. Grape pomace compost harbors organohalide-respiring *Dehalogenimonas* species with novel reductive dehalogenase genes. *ISME J.* **2017,** *11*, 2767-2780.

73. Molenda, O.; Quaile, A. T.; Edwards, E. A. *Dehalogenimonas* sp. strain WBC-2 genome and identification of its *trans*-dichloroethene reductive dehalogenase, TdrA. *Appl. Environ. Microbiol.* **2016,** *82*, 40-50.

74. Trueba-Santiso, A.; Wasmund, K.; Soder-Walz, J. M.; Marco-Urrea, E.; Adrian, L. Genome sequence, proteome profile, and identification of a multiprotein reductive dehalogenase complex in *Dehalogenimonas alkenigignens* strain BRE15M. *J. Proteome Res.* **2021,** *20*, 613-623.

75. Schumacher, W.; Holliger, C.; Zehnder, A. J.; Hagen, W. R. Redox chemistry of cobalamin and iron-sulfur cofactors in the tetrachloroethene reductase of *Dehalobacter restrictus*. *FEBS Lett.* **1997,** *409*, 421-425.

76. Goris, T.; Schubert, T.; Gadkari, J.; Wubet, T.; Tarkka, M.; Buscot, F.; Adrian, L.; Diekert, G. Insights into organohalide respiration and the versatile catabolism of *Sulfurospirillum multivorans* gained from comparative genomics and physiological studies. *Environ. Microbiol.* **2014,** *16*, 3562-3580.

77. Nonaka, H.; Keresztes, G.; Shinoda, Y.; Ikenaga, Y.; Abe, M.; Naito, K.; Inatomi, K.; Furukawa, K.; Inui, M.; Yukawa, H. Complete genome sequence of the dehalorespiring bacterium *Desulfitobacterium hafniense* Y51 and comparison with *Dehalococcoides ethenogenes* 195. *J. Bacteriol.* **2006,** *188*, 2262-2274.

78. Gerritse, J.; Renard, V.; Pedro Gomes, T. M.; Lawson, P. A.; Collins, M. D.; Gottschal, J. C. *Desulfitobacterium* sp. strain PCE1, an anaerobic bacterium that can grow by reductive dechlorination of tetrachloroethene or *ortho*-chlorinated phenols. *Arch. Microbiol.* **1996,** *165*, 132-140.

79. Kim, S.-H.; Harzman, C.; Davis, J. K.; Hutcheson, R.; Broderick, J. B.; Marsh, T. L.; Tiedje, J. M. Genome sequence of *Desulfitobacterium hafniense* DCB-2, a Gram-positive anaerobe capable of dehalogenation and metal reduction. *BMC Microbiol.* **2012,** *12*, 21.

80. Löffler, F. E.; Sanford, R. A.; Tiedje, J. M. Initial characterization of a reductive dehalogenase from *Desulfitobacterium chlororespirans* Co23. *Appl. Environ. Microbiol.* **1996,** *62*, 3809-3813.

81. Chen, K.; Huang, L.; Xu, C.; Liu, X.; He, J.; Zinder, S. H.; Li, S.; Jiang, J. Molecular characterization of the enzymes involved in the degradation of a brominated aromatic herbicide. *Mol. Microbiol.* **2013,** *89*, 1121-1139.

82. Tront, J. M.; Amos, B. K.; Löffler, F. E.; Saunders, F. M. Activity of *Desulfitobacterium* sp. strain Viet1 demonstrates bioavailability of 2,4-dichlorophenol previously sequestered by the aquatic plant Lemna minor. *Environ. Sci. Technol.* **2006,** *40*, 529-535.

83. Boyer, A.; Pagé-BéLanger, R.; Saucier, M.; Villemur, R.; Lépine, F.; Juteau, P.; Beaudet, R. Purification, cloning and sequencing of an enzyme mediating the reductive dechlorination of 2,4,6-trichlorophenol from *Desulfitobacterium frappieri* PCP-1. *Biochem. J.* **2003,** *373*, 297-303.

84. Gauthier, A.; Beaudet, R.; Lépine, F.; Juteau, P.; Villemur, R. Occurrence and expression of crdA and cprA5 encoding chloroaromatic reductive dehalogenases in *Desulfitobacterium* strains. *Can. J. Microbiol.* **2006,** *52*, 47-55.

85. Halliwell, T.; Fisher, K.; Rigby, S. E. J.; Leys, D. Heterologous production and biophysical characterization of catabolic *Nitratireductor pacificus* pht-3B reductive dehalogenase. *Methods Enzymol.* **2022,** *668*, 327-347.

86. Neumann, A.; Wohlfarth, G.; Diekert, G. Tetrachloroethene dehalogenase from *Dehalospirillum multivorans*: cloning, sequencing of the encoding genes, and expression of the *pceA* gene in *Escherichia coli*. *J. Bacteriol.* **1998,** *180*, 4140-4145.

87. Buttet, G. F.; Holliger, C.; Maillard, J. Functional genotyping of *Sulfurospirillum* spp. in mixed cultures allowed the identification of a new tetrachloroethene reductive dehalogenase. *Appl. Environ. Microbiol.* **2013,** *79*, 6941-6947.

88. Alfán-Guzmán, R.; Ertan, H.; Manefield, M.; Lee, M. Isolation and characterization of *Dehalobacter* sp. strain TeCB1 including identification of TcbA: a novel tetra- and trichlorobenzene reductive dehalogenase. *Front. Microbiol.* **2017,** *8*, 558.

89. Molenda, O.; Tang, S.; Edwards, E. A. Complete genome sequence of *Dehalococcoides mccartyi* strain WBC-2, capable of anaerobic reductive dechlorination of vinyl chloride. *Genome Announc.* **2016,** *4*.

90. Chai, B.; Li, X.; Liu, H.; Lu, G.; Dang, Z.; Yin, H. Bacterial communities on soil microplastic at Guiyu, an E-Waste dismantling zone of China. *Ecotoxicol. Environ. Saf.* **2020,** *195*, 110521.

91. Chen, J.; Bowman, K. S.; Rainey, F. A.; Moe, W. M. Reassessment of PCR primers targeting 16S rRNA genes of the organohalide-respiring genus *Dehalogenimonas*. *Biodegradation* **2014,** *25*, 747-756.

92. Ritalahti, K. M.; Amos, B. K.; Sung, Y.; Wu, Q.; Koenigsberg, S. S.; Löffler, F. E. Quantitative PCR targeting 16S rRNA and reductive dehalogenase genes simultaneously monitors multiple *Dehalococcoides* strains. *Appl. Environ. Microbiol.* **2006,** *72*, 2765-2774.
